# Unveiling the Multielectron Acceptor Properties of π-Expanded Pyracylene: Reversible Boat to Chair Conversion

**DOI:** 10.1021/jacs.4c02314

**Published:** 2024-05-14

**Authors:** Yikun Zhu, Jan Borstelmann, Oliver Bertleff, John Bergner, Zheng Wei, Christian Neiss, Andreas Görling, Milan Kivala, Marina A. Petrukhina

**Affiliations:** † Department of Chemistry, University at Albany, State University of New York, Albany, New York 12222, United States; ‡ Organisch-Chemisches Institut, Universität Heidelberg, Im Neuenheimer Feld 270, Heidelberg 69120, Germany; § Lehrstuhl für Theoretische Chemie, Friedrich-Alexander Universität Erlangen-Nürnberg (FAU), Egerlandstraße 3, Erlangen 91058, Germany; ∥ Erlangen National High Performance Computing Center (NHR@FAU), Martensstr. 1, Erlangen 91058, Germany

## Abstract

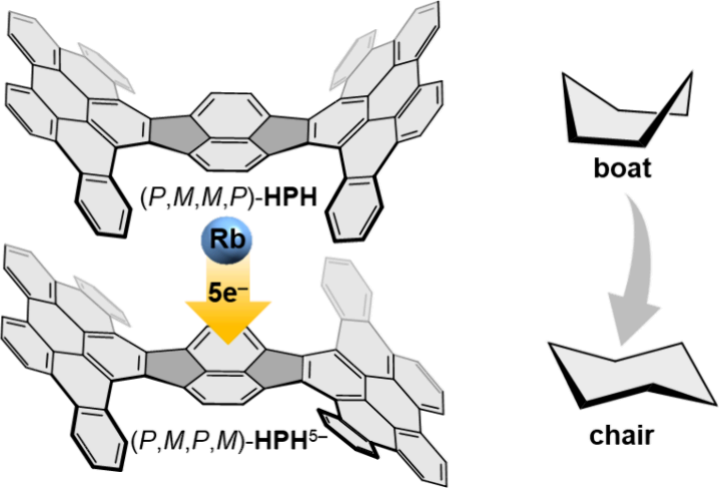

In this work, the chemical reduction of a hybrid pyracylene-hexa-*peri*-hexabenzocoronene (**HPH**) nanographene was investigated with different alkali metals (Na, K, Rb) to reveal its remarkable multielectron acceptor abilities. The UV–vis and ^1^H NMR spectroscopy monitoring of the stepwise reduction reactions supports the existence of all intermediate reduction states up to the hexaanion for **HPH**. Tuning the experimental conditions enabled the synthesis of the **HPH** anions with gradually increasing reduction states (up to −5) isolated with different alkali metal ions as crystalline materials. The single-crystal X-ray diffraction structure analysis demonstrates that the highly negatively charged **HPH** anions (−4 and −5) exhibit a drastic geometry change from boat-shaped (observed in the neutral parent, mono- and dianions) to a chair conformation, which was proved to be fully reversible by NMR spectroscopy. DFT calculations show that this geometry change is induced by an enhanced interaction between the coordinated metal ions and negatively charged **HPH** core in the chair conformation.

## Introduction

Polycyclic aromatic hydrocarbons (PAHs) serve as redox-active materials in various applications.^1−5^ Their optoelectronic and structural properties can be finely tuned by incorporation of heteroatoms or nonhexagonal rings.^6−10^ By incorporation of a 5-membered ring, positive curvature (bowl shape) can be induced.^11^ This leads to a considerable pyramidalization of the sp^2^ carbon atoms within the polycyclic framework, thereby weakening the π bonds and reducing the energy of the lowest unoccupied molecular orbital (LUMO).^12−14^ Moreover, according to Hückel’s rule, the conjugated 5-membered ring can take up one electron to achieve formal aromaticity, which makes these compounds prone to reduction.^15−17^

The most prominent example of this behavior is found in the C_60_-fullerene, which incorporates 12 5-membered rings, leading to a spherical geometry.^18−20^ C_60_ can reversibly take up six electrons, successively leading to the corresponding hexaanion C_60_^6^^–^.^21,22^ This propensity for multielectron reduction has led to the prominence of C_60_ as an electron acceptor in specific applications. In particular, salts resulting from the reduction of C_60_ with alkali metals have gained increasing attention due to their potential use as energy storage and superconducting materials.^23−27^

To harness these notable electron-accepting properties, PAHs representing defined fullerene cutouts such as bowl-shaped corannulene or sumanene have emerged as attractive synthetic targets.^28−37^ The bowl-shaped geometry makes them particularly prone to reduction/deprotonation and complexation with alkali metals.^38−41^ Even the smallest buckybowl, corannulene, exhibits high electron-accepting ability and can take up to four electrons, forming a set of carbanions with selective concave and convex metal binding.^41−44^ Several examples of bowl-shaped acceptors and their derivatives have been reported recently,^45−47^ although most of them can readily accept two electrons, while the analysis of highly reduced PAHs by X-ray crystallography remains challenging.^48,49^

Furthermore, another conceivable, yet not less appealing, fullerene cutout, namely, pyracylene, remains considerably less explored.^50−53^ With its 12π-electron periphery, pyracylene represents a formally antiaromatic cutout of C_60_.^54−57^ Upon addition of two electrons, two cyclopentadienyl moieties are formed, which render the resulting dianion with its 14π electrons formally aromatic. Nonetheless, pyracylene and its homologue dibenzopyracylene show a planar geometry.^58,59^ Thereby, they cannot benefit from the pyramidalization and strong metal-binding ability to further enhance their electron-accepting properties as observed for buckybowls.^60^

To address this limitation, we have recently developed a hybrid pyracylene-hexa-*peri*-hexabenzocoronene (HBC) abbreviated as **HPH**.^53^ This compound adopts a boat-like geometry both in solution and in the solid-state with a bowl depth of 3.79 Å, resulting in a curved pyracylene core with pyramidalized sp^2^ carbon atoms. Cyclic voltammetry measurements of **HPH** revealed two reversible reductions at −1.46 and −1.82 V (vs ferrocene/ferrocenium (Fc/Fc^+^)) in THF. This points toward better charge stabilization compared to pristine pyracylene, which is considerably more difficult to reduce at −1.56 and −2.14 V.^53,61,62^ Furthermore, **HPH** also displays enhanced reducibility relative to other curved π-expanded nanographenes from the literature, such as the warped nanographene (with three reductions at −1.59, −2.02, and −2.31 V vs Fc/Fc^+^) or the corannulene-embedded [6]helicene (with two reductions at −1.90 and −2.18 V vs Fc/Fc^+^).^63,64^

**Figure 1:**
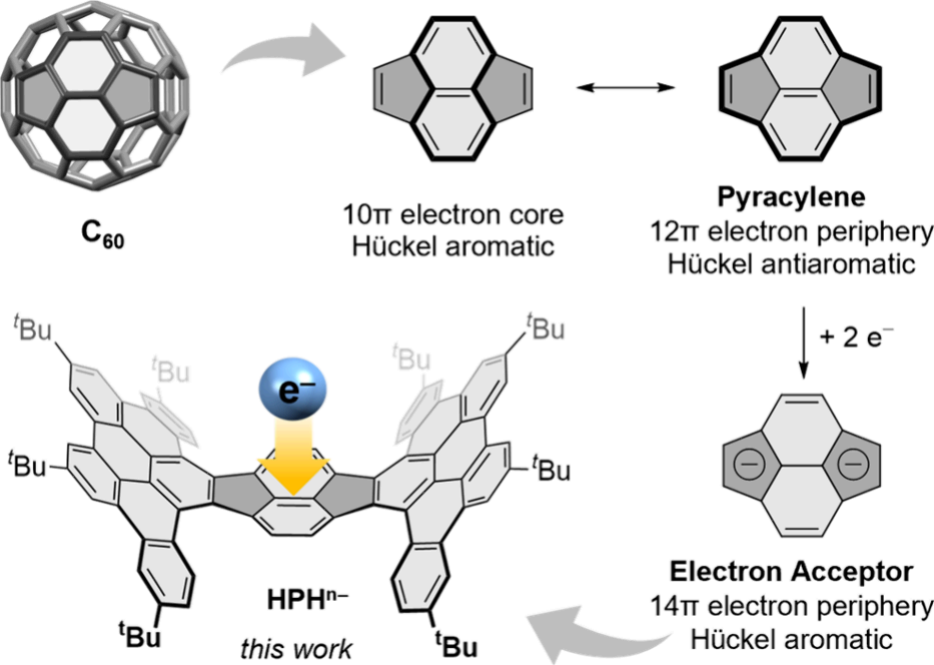
Planar pyracylene represents a cutout of fullerene C_60_ with pronounced electron-accepting properties. π-Expansion induces curvature into the pyracylene core and leads to the boat-shaped hybrid-pyracylene-HBC (**HPH**)^53^ with potentially enhanced electron-accepting properties explored in this work.

The enhanced electron-accepting properties and bowl-shaped geometry of **HPH** prompted us to investigate its chemical reduction behavior with alkali metals and to target isolation of the gradually reduced negatively charged products in the presence of different alkali metal counterions (Figure 1).

## Results and Discussion

### Chemical Reduction and Crystallization of HPH Anions

**Figure 2:**
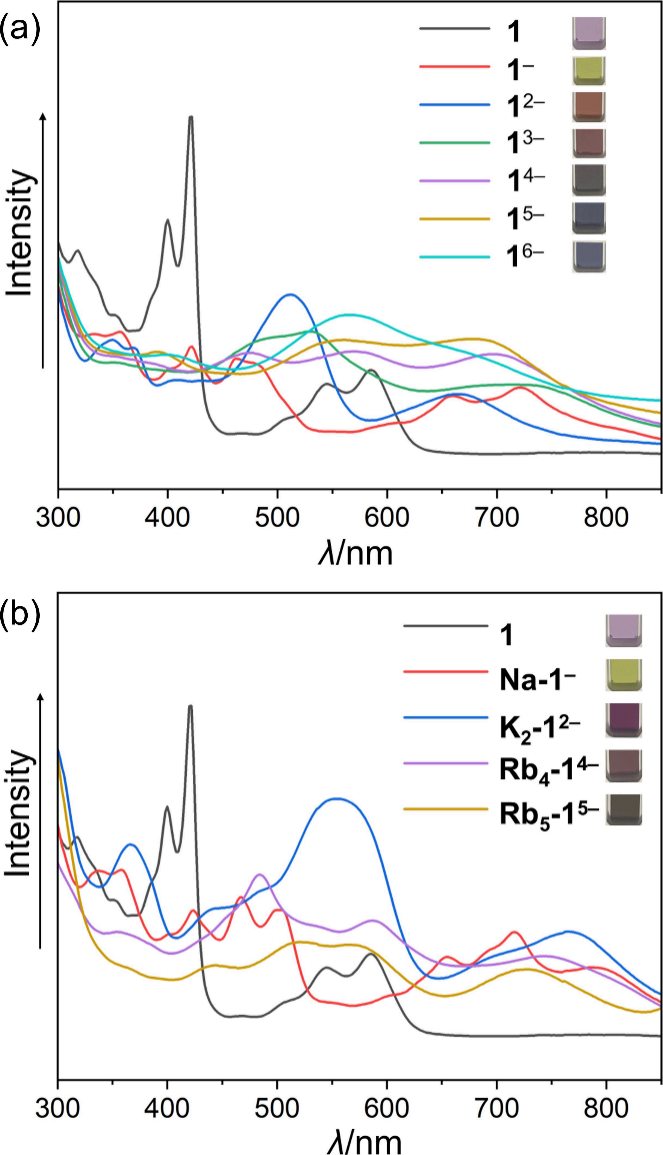
UV–vis spectra of (a) **HPH** and its *in situ* generated anions and (b) crystals of **1**, **Na-1**^**–**^, **K**_**2**_**-1**^**2–**^, **Rb**_**4**_**-1**^**4–**^, and **Rb**_**5**_**-1**^**5–**^ dissolved in THF.

To probe the reduction limits, UV–vis spectroscopy was used to monitor the reaction color changes for the stepwise chemical reduction of **HPH** (C_102_H_96_, **1**). The addition of Rb metal to a THF solution of **1** led to a quick color change from purple (color of the neutral parent) to grass green (monoanion), reddish-purple (dianion), brownish purple (trianion), dark purple (tetraanion), and violet/blue color for penta- and hexa-reduced products (Figure 2a, see the Supporting Information for more details). To verify these experimental observations of potential 6-fold reduction, *in situ*^1^H NMR and EPR spectroscopy measurements were carried out and provided strong support for remarkable six-electron-accepting abilities of **HPH** (Figures S12 and S9). The *in situ* generated dianion, tetraanion, and hexaanion were successfully detected by ^1^H NMR spectroscopy, while the intermediate NMR silent radicals (−1, −3, and −5) were identified by EPR spectroscopy. The *in situ* EPR study of **HPH** reduction with Rb metal clearly revealed four EPR “silent” diamagnetic species (including neutral **HPH**) and three EPR detectable radicals. The complex structure of this PAH and limitation of the instrument prevented detailed analysis of multiplicity or hyperfine coupling, but important information can be extracted. In the spectra of both mono- and trianion radicals, a clear set of multiplet peaks are observed in contrast to a broad peak of the penta-reduced state. This may indicate the rapid exchange between the unpaired electrons for the latter highly charged product. Based on NMR results, a similar exchange between diamagnetic and paramagnetic species can be observed, and the *in situ* generated products with charges of 0, −2, −4, and −6 offered four distinct NMR patterns. The chemical shifts of aromatic protons in ^1^H NMR spectra of neutral **HPH** and its dianion show up in a close range (7.90–9.43 ppm for **HPH** vs 7.75–9.85 ppm in the dianion). However, upon further reduction to the tetraanion and hexaanion, the aromatic protons become largely upfield shifted to 4.66–6.54 ppm for **HPH**^4–^ and 4.42–6.15 ppm for **HPH**^6–^, thus indicating significant electron density rearrangement. Importantly, the NMR spectrum of the hexa-reduced product quenched with O_2_ (Figure S12) reveals the reaction reversibility and points out the stability and inherent flexibility of the **HPH** core toward redox processes.

**Scheme 1:**
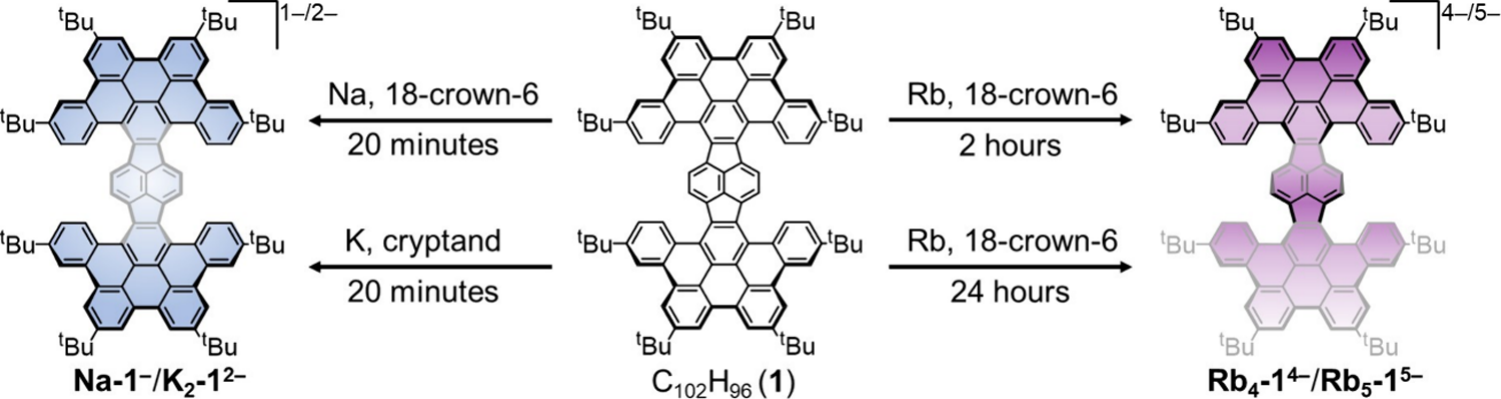
Chemical Reduction of **1**

Following the spectroscopic study, the chemical reduction of **1** was carried out with several alkali metals ranging in size and coordination preferences and using anhydrous solvents and inert atmosphere conditions. By changing the nature of alkali metals (Na, K and Rb) and secondary ligands (18-crown-6 ether and [2.2.2]cryptand), the successful isolation of several gradually reduced products with distinct colors (Figure 2b) has been achieved. The use of limited reaction time coupled with addition of crown ether in the Na-induced reaction allowed the isolation of a singly reduced product, [Na^+^(18-crown-6)(THF)_2_][**1**^–^] (**Na-1**^**–**^), crystallized as a solvent-separated ion product (SSIP) with four interstitial THF molecules as [**Na-1**^**–**^]·4THF. The addition of [2.2.2]cryptand facilitated isolation of the doubly reduced product with potassium counterions, [K^+^(cryptand)]_2_[**1**^2–^] (**K**_**2**_**-1**^**2–**^), crystallized as a SSIP with four interstitial hexane molecules as [**K**_**2**_**-1**^**2–**^]·4C_6_H_14_. Notably, the use of prolonged reaction time for the Na- and K-induced reduction of **HPH** leads to additional solution color changes indicative of further reduction (Figures S1 and S2). However, these reactions failed to produce single-crystalline products. The switch to Rb metal and longer reaction times enabled the formation of the tetra-reduced anion isolated as a contact-ion complex with rubidium countercations, as [{Rb^+^(18-crown-6)}_4_(**1**^4–^)]·10THF ([**Rb**_**4**_**-1**^**4–**^]·10THF). The extended reaction time (24 h) for this system resulted in the isolation of the penta-reduced product, [Rb^+^(18-crown-6)][{Rb^+^(18-crown-6)}_4_(**1**^5–^)]·5THF ([**Rb**_**5**_**-1**^**5–**^]·5THF, Scheme 1). Notably, the *in situ* UV–vis spectroscopy monitoring of the **HPH** reaction with Rb metal in the presence of 18-crown-6 ether illustrated that, under these conditions, the reaction stops at the penta-reduced state (Figure S3). Unfortunately, our numerous attempts to isolate the products of the triply- and hexa-reduced states of **HPH** were not successful, as the resulting very thin fibrous-like needles did not provide sufficient diffraction for structure solution in both cases.

### Crystal Structures of Stepwise-Reduced HPH Products

**Figure 3:**
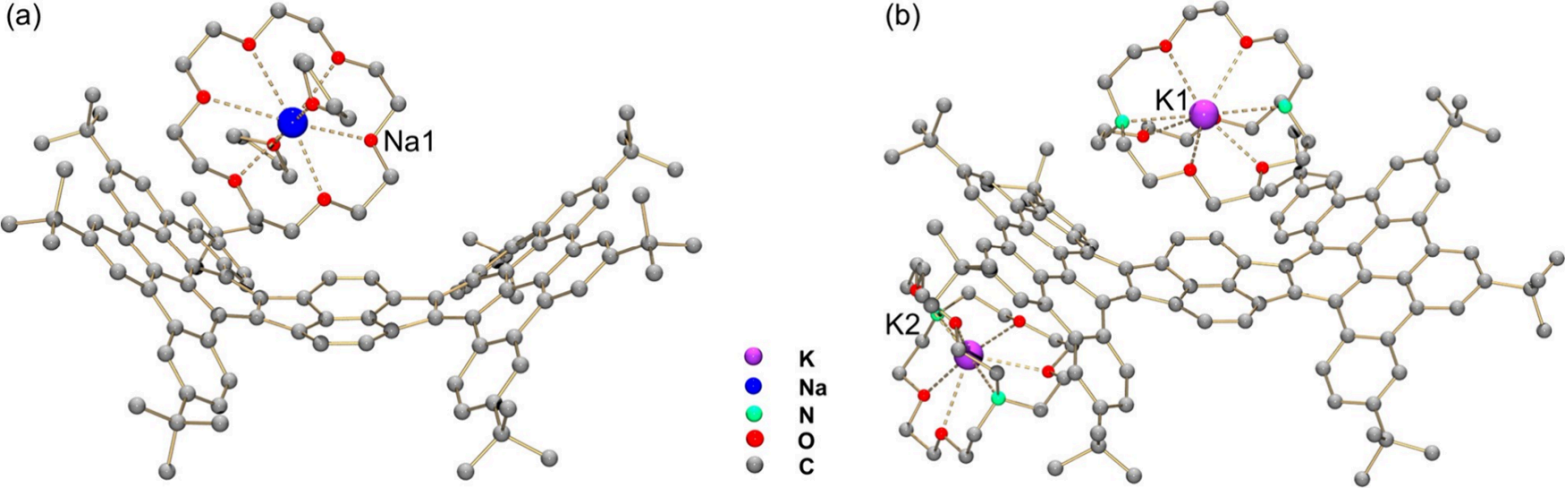
Crystal structures of (a) **Na-1**^**–**^ and (b) **K**_**2**_**-1**^**2–**^, ball-and-stick model. Hydrogen atoms are omitted for clarity. The Na···O_crown_ and Na···O_THF_ distances range over 2.329(5)–2.998(15) and 2.286(9)–2.656(6) Å, respectively. The K···N_cryptand_ distances and K···O_cryptand_ distances are 2.981(5)–3.020(5) and 2.735(3)–2.878(4) Å, respectively.

The stepwise reduction of **1** with Na and K metals provided two SSIPs with different low charges (−1 and −2) of the **HPH** core, namely, **Na-1**^**–**^ and **K**_**2**_**-1**^**2–**^. In the crystal structure of **Na-1**^**–**^, two crystallographically independent molecules are found with similar geometric parameters (Figure S14), and only one of which is discussed below. The Na^+^ ion entrapped by one 18-crown-6 and two THF molecules has no direct contacts with the anion **1**^–^ (Figure 3a), with the Na···O_crown_ and Na···O_THF_ distances comparable to the previously reported values.^49,46^ In the doubly reduced product **K**_**2**_**-1**^**2–**^, the two K^+^ ions wrapped by one [2.2.2]cryptand each remain separated from the dianion, with K···N_cryptand_ and K···O_cryptand_ distances in line with the values reported in the literature (Figure 3b).^65,66^

**Figure 4:**
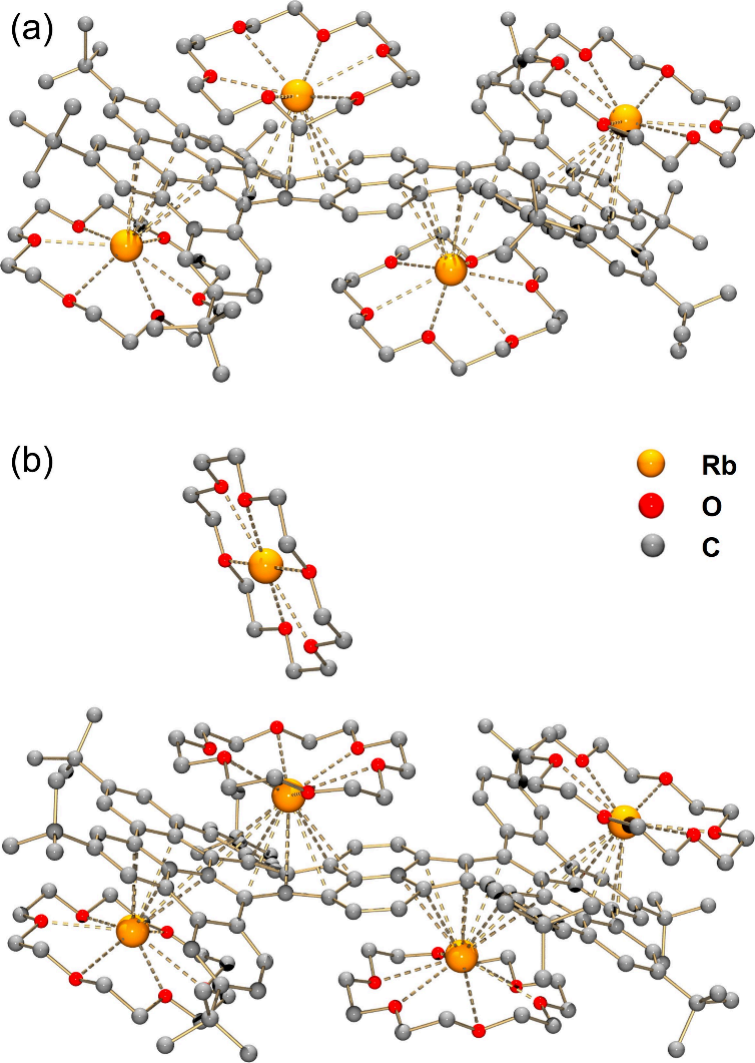
Crystal structures of (a) **Rb**_**4**_**-1**^**4–**^ and (b) **Rb**_**5**_**-1**^**5–**^, ball-and-stick model. Hydrogen atoms are omitted for clarity. The Rb···O_crown_ distances for **Rb**_**4**_**-1**^**4–**^ and **Rb**_**5**_**-1**^**5–**^ are in the ranges of 2.853(3)–3.144(2) Å and 2.790(3)–3.154(14) Å, respectively. All Rb···O_crown_ distances are comparable to the reported values.^47,67^

The reduction reaction of **1** with Rb metal provided access to the highly reduced **HPH** anions. The addition of 18-crown-6 ether facilitated crystallization of two contact-ion products with high negative charges (−4 and −5), **Rb**_**4**_**-1**^**4–**^ and **Rb**_**5**_**-1**^**5–**^. In the crystal structure of **Rb**_**4**_**-1**^**4–**^ (Figure 4a), the highly charged tetraanion converted from a boat-shape conformation to a recliner-chair-type structure, with molecular symmetry changed from *C*_2*v*_ to *C*_2*h*_. The four Rb^+^ ions are bound to the **HPH**^**4–**^ core, two of which nest in the concave cavities of the core with the other two bound to the HBC units from the opposite sides of the nanographene surface (Figure 6a,c). The Rb–C distances in **Rb**_**4**_**-1**^**4–**^ spanning over 3.136(2)–3.646(3) Å are comparable to the range (3.025(12)–3.640(12) Å) found in the tetra-reduced corannulene product with mixed Li/Rb metals.^42^ Each Rb^+^ ion is additionally capped by an 18-crown-6 molecule from an open end.

Extending the reaction time to 24 h (Scheme 1) with Rb metal allowed the isolation of the remarkable penta-reduced product, **Rb**_**5**_**-1**^**5–**^ (Figure 4b). In addition to four-coordinated Rb^+^ ions, the fifth Rb^+^ ion, wrapped by an 18-crown-6 molecule, resides as a solvent-separated counterion. Compared to **Rb**_**4**_**-1**^**4–**^, the increased negative charge of **1**^**5–**^ leads to a tighter and more symmetrical coordination of Rb^+^ ions in **Rb**_**5**_**-1**^**5–**^ (Figure 6b), with the Rb–C distances notably reduced to 3.071(2)–3.598(2) Å (Table S2).

In the solid-state structure of **Na-1**^**–**^, one [Na^+^(18-crown-6)(THF)_2_] cationic moiety is wrapped by the concave faces of two **1**^–^ anions with C–H···π interactions of 2.601(7)–2.990(7) Å. Through additional C–H···π interactions between the other cationic moiety and convex face of **1**^–^ (2.584(7)–3.010(7) Å), an extended 1D column is formed (Figure S20). A similar packing pattern is observed in **K**_**2**_**-1**^**2–**^, namely, two [K^+^(cryptand)] moieties are wrapped by two anions with the C–H···π interactions of 2.362(7)–3.041(7) Å. The other two cationic moieties bridge two **HPH** units with C–H···π interactions of 2.508(7)–2.978(7) Å to form a 1D column (Figure S21). In the **Rb**_**4**_**-1**^**4–**^ and **Rb**_**5**_**-1**^**5–**^ products, no notable secondary interactions are observed in the solid-state structures.

### Charge-Dependent Core Deformation

**Figure 5:**
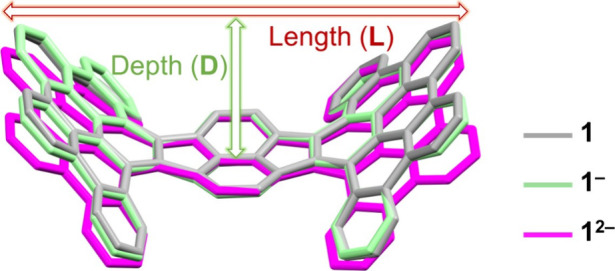
Core deformation in **1**, **1**^**–**^, and **1**^**2–**^.

The isolation of the “naked” monoanion and dianion of **HPH** enables the evaluation of structural deformation upon addition of one and two electrons but without direct alkali metal-binding influence (*vide supra*). In comparison to neutral parent **1**, the monoanion preserves the boat-shaped geometry with a small decrease of curvature, as shown by the slightly increased molecular length (**L**, 15.734(7) Å vs 15.558 Å in **1**) and notably reduced ligand height (**H**, 9.724(7) Å vs 10.116 Å in **1**) and bowl depth (**D**, 3.748(7) Å vs 3.787 Å in **1**), accompanied by changes in dihedral angles (Table 1). The addition of the second electron to **1** further flattens its carbon backbone. The geometric parameters of **1**^2–^ clearly illustrate the reduced curvature, with the height-to-length ratio decreased to 50.7 vs 65.0% in the neutral parent and 61.8% in the monoanion. The boat-type structure of **1**^2–^ is found to be significantly shallower (depth of 2.861(7) Å, Figure 5) than in **1** and **1**^–^. Notably, the 2-fold reduction also largely decreases the helical torsion from 50° in **1** and **1**^–^ to 43° in **1**^2–^ (Table 1).

**Table 1:**
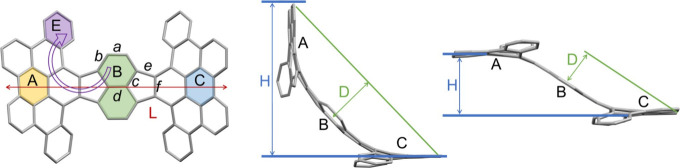
Selected Distances (Å) and Angles (deg) in **1**, **1^–^**, **1^2–^**, **1^4–^**, and **1^5–^**, along with a Labeling Scheme

parameter	**1**	**1**^**–**^	**1**^**2–**^	**1**^**4–**^	**1**^**5–**^
***a***	1.429/1.434	1.386(6)/1.398(6)	1.380(7)/1.383(7)	1.385(4)	1.383(3)
***b***	1.370–1.385	1.398(6)–1.415(6)	1.402(7)–1.428(7)	1.440(4)/1.448(4)	1.426(3)/1.440(3)
***c***	1.399–1.406	1.401(6)–1.433(6)	1.383(7)–1.422(7)	1.416(4)	1.403(3)/1.415(3)
***d***	1.381	1.373(6)	1.350(7)	1.385(5)	1.370(4)
***e***	1.493–1.500	1.457(6)–1.471(6)	1.421(7)–1.462(7)	1.442(4)/1.445(4)	1.436(3)/1.439(3)
***f***	1.401/1.414	1.427(6)/1.446(6)	1.440(7)/1.448(7)	1.475(4)	1.451(3)
length (**L**)	15.558	15.734(7)	16.470(7)	16.636(5)	16.496(4)
height (**H**)	10.116	9.724(7)	8.350(7)	3.685(5)	4.801(4)
depth (**D**)	3.787	3.748(7)	2.861(7)	2.767(5)	2.804(4)
∠A/B	42.6	41.8(5)	30.8(5)	29.9(4)	30.0(3)
∠A/C	79.8	75.4(5)	61.0(5)	0.0(4)	0.0(3)
∠B/C	37.3	33.6(5)	30.6(5)	29.9(4)	30.0(3)
helical torsion (∠B/E)	50.6	50.5(5)	43.2(5)	41.4(4)	42.0(3)

Notably, in both singly and doubly reduced products, the C–C bonds around the 5-membered rings of the pyracylene core experience a distinct bond length change and rearrangement. The bonds ***e*** become significantly shorter than in the neutral parent, while the bonds ***f*** become longer (Table 1). Similar bond length alterations were observed for the doubly reduced products of phenylenetetracene and indenocorannulene.^68,47^ The bond lengths of ***a*** and ***d*** become smaller and more equalized (1.43/1.38 Å in **1** vs 1.39/1.37 Å in **1**^**–**^ and 1.38/1.35 Å in **1**^**2–**^). In contrast, the C–C bonds ***b*** and ***c*** are slightly elongated and cover a broader range (Table 1).

**Figure 6:**
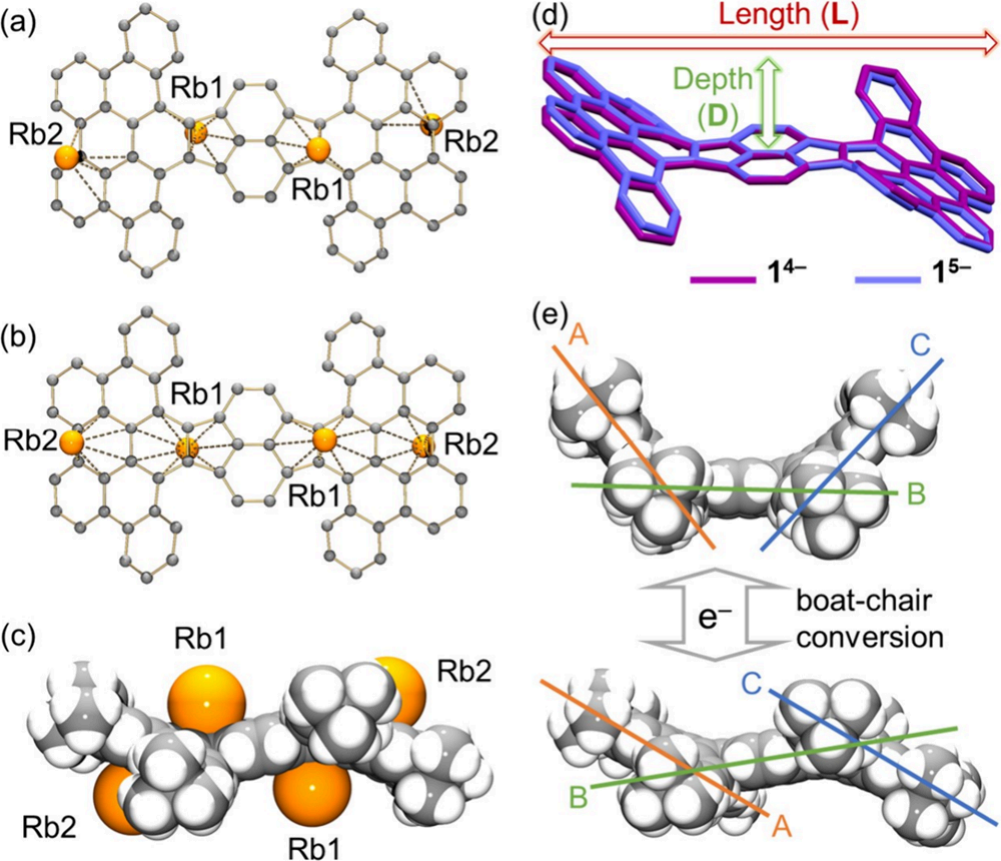
Metal coordination in (a) **Rb**_**4**_**-1**^**4–**^ and (b) **Rb**_**5**_**-1**^**5–**^, ball-and-stick model. Hydrogen atoms, ^*t*^Bu-groups, and 18-crown-6 are omitted for clarity. (c) Coordination of Rb^+^ ions, space-filling model; 18-crown-6 molecules are omitted. (d) Curvature difference between **1**^**4–**^ and **1**^**5–**^. (e) Charge-dependent boat–chair conversion between **1** (top) and **1**^**4–**^/**1**^**5–**^ (bottom), space-filling models.

In **Rb**_**4**_**-1**^**4–**^ and **Rb**_**5**_**-1**^**5–**^, the high negative charge of **HPH** makes it highly favored for metal coordination, which stabilizes the system by mitigating the charge and electron density. To accommodate coordination of multiple Rb^+^ ions, the boat-shaped **HPH** is converted into a chair-type geometry. The reduction-induced boat–chair conversion largely increases the molecular length (16.636(5)/16.496(4) Å in **1**^**4–**^/**1**^**5–**^ vs 15.558 Å in **1**) and reduces the bowl depth (2.767(5)/2.804(4) Å in **1**^**4–**^/**1**^**5–**^ vs 3.787 Å in **1**) of the **HPH** framework (Figure 6d). The same trend is observed for the decreased dihedral angles between planes A, B, and C (Figure 6e), indicating the overall reduced curvature of the carbon scaffold.

The similarity of metal coordination patterns in **Rb**_**4**_**-1**^**4–**^ and **Rb**_**5**_**-1**^**5–**^ enables the evaluation of the stepwise charge increase on the **HPH** geometry. Compared to the tetraanion, the addition of the fifth electron shortens the C–C bond distances along the whole carbon core (Table S4). As a result, the overall molecular length is reduced (16.496(4) Å in **1**^**5–**^ vs 16.636(5) Å in **1**^**4–**^). In contrast, the height (distance between the A and C planes, see Table 1 insert) of the pentaanion is largely increased to 4.801(4) Å from 3.685(5) Å in **1**^**4–**^. In addition, the bowl depth and helical torsion are also slightly increased (Table 1).

In summary, the controlled electron addition at the low reduction level allows the **HPH** anions to keep the same boat-shaped geometry with reduced curvature and increased planarity. In contrast, further reduction of **HPH** (beyond tetraanion) favors a chair-type conformation, with alkali metal counterions binding to the concave cavities and side HBC units on both sides of the carbon backbone. Following the boat–chair conversion, the acquisition of an additional fifth electron only slightly increases the curvature of the pentaanion core.

The poor solubility of **Rb**_**4**_**-1**^**4–**^ and the radical character of **Rb**_**5**_**-1**^**5–**^ thwarted the NMR spectroscopy investigation of the effect of a secondary 18-crown-6 ligand. Therefore, UV–vis spectroscopy investigation was used for getting further insights. A comparison between the *in situ* UV–vis spectra of [**HPH**/Rb/18-crown-6] (Figure S3) and [**HPH**/Rb] in THF (Figure S4) leads to the following observations: (1) the addition of 18-crown-6 contributes to an unexpected stability of the pentaanion, in contrast to the clear conversion to the hexaanion in the absence of crown ether. A similar crown ether-assisted stabilization of the singly reduced radical species was observed for the planar coronene anion,^69^ while examples for highly reduced systems have not been reported previously. (2) Besides the lack of the hexa-reduced stage, discrepancies can be observed in the absorbance spectra of the highly negatively charged tetraanion and pentaanion (*vide infra*).

### Computational Analysis

A computational study on the density functional theory (DFT) level of theory was conducted to further analyze the electron-accepting behavior of **HPH** (see the Supporting Information for more details). To separate the influences of Rb^+^ and the 18-crown-6 ligand, we first considered the different reduction states of “naked” **1**^*n*–^ (*n* = 0–6) embedded in a polarizable continuum mimicking THF. As indicated by the crystal structures, **HPH** can essentially adopt two different conformations, the boat (like in the crystal structures of neutral **1** and **Na-1**^**–**^ and **K**_**2**_**-1**^**2–**^) and the chair (as found for **Rb**_**4**_**-1**^**4–**^ and **Rb**_**5**_**-1**^**5–**^) conformation. In the absence of stabilizing Rb^+^ ions, the boat conformation is always slightly more stable by ∼2 kcal/mol (Table S5, upper row). The boat–chair interconversion occurs via an intermediate state (∼5 kcal/mol less stable than the boat conformation) and has an activation barrier of ≲13 kcal/mol, indicating a rapid conformational change in solution at room temperature (Table S6, Figure S22). Each reduction state is stable in the sense that all electrons are bound, i.e., all occupied orbitals have negative energy with respect to a free electron (Table S7), which highlights the large electron-accepting capabilities of **HPH**. By inspecting the electron density differences between the charged and neutral species, one notices that the first two electrons are mostly localized at the pyracylene core, whereas further electron addition involves the extended π-system of **HPH** (Figure S23). Moreover, the bowl depth of “naked” **1**^*n*–^ decreases up to 2-fold reduction and then increases again to reach a nearly constant value of 3.0 Å (Table S9).

Interestingly, the coordination of {Rb^+^(18-crown-6)} ions to **1**^*n*–^ switches stabilities, causing the chair conformation to become more stable by ∼3 kcal/mol (Table S5, bottom). To better understand what exactly causes the change of stability, we evaluated the energy differences between the two conformations at the frozen geometries of {Rb^+^(18-crown-6)}_4_(**1**^*n*–^) with either the crown ethers only or the entire {Rb^+^(18-crown-6)} moieties removed (Table S8). While the boat conformation is more stable in the uncomplexed form, the placement of the Rb^+^ ions at the coordination sites corresponding to the crystal structure {Rb^+^(18-crown-6)}_4_(**1**^*n*–^) favors the chair conformation (Table S8). However, a complete relaxation of the Rb^+^_4_(**1**^*n*–^) system essentially leads to a shift of the Rb^+^ ions and shows that they prefer positions closer to the pyracylene core (Figure S24), where the negative charge density is higher. Moreover, the geometric rearrangement of Rb^+^ largely annihilates the energetic preference of the chair conformation (Table S5, middle row). In {Rb^+^(18-crown-6)}_4_(**1**^*n*–^), however, two Rb^+^ cations can stay close to the pyracylene core in the chair conformation, whereas in the boat conformation, none of the Rb^+^ ions can interact with the pyracylene core (Figure S25). This suggests that the chair conformation of {Rb^+^(18-crown-6)}_4_(**1**^*n*–^) is more stable because the Rb^+^ cations can occupy more favorable binding sites. Therefore, the observed geometry change from boat to chair is not intrinsically driven, but due to optimized interactions between the {Rb^+^(18-crown-6)} moiety and **HPH** anion.

Experimentally, it was found that the reduction of **HPH** without 18-crown-6 yields the hexaanion, whereas in the presence of the crown ether, the reaction stops at the pentaanion. To analyze this finding, let us consider the formation of the hexaanion (in THF) in both cases:
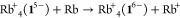
1
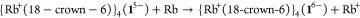
2

From the energies of the involved anions, one can easily compute the energy difference between these two reactions (without the need to calculate the energies of metallic Rb and solvated Rb^+^), which yields that the reduction of the pentaanion with bare Rb (in absence of the crown ether) is favored by ∼8 kcal/mol, i.e., the hexaanion is better stabilized by bare Rb^+^ ions than by {Rb^+^(18-crown-6)}.

Furthermore, the UV–vis absorption spectra of the *in situ* generated **HPH** anions were compared with the simulated (on the TD-DFT level) spectra of the **1**^*n*–^ series (Figure S26). The experimental spectra, especially those for the lower reduced states up to the trianion, are well reproduced confirming the previously made assignment of the spectra. Moreover, all reduced species exhibit electronic transitions in the near-infrared (NIR) region and, partly, even lower. These low-lying excitations correspond to π–π transitions; the same applies for the low-lying transitions in the visible region.

For the trianion and higher charged **HPH**, we also considered the effect of the π-interacting Rb^+^ cations (Figure S26). In case of the trianion, two Rb^+^ ions, for **1**^*n*–^ (*n* = 4–6) four Rb^+^, were taken into account (geometries were fully relaxed). As expected, coordination of Rb^+^ causes some shifts and intensity changes of the absorption bands, but the changes are overall moderate. The conformation or exact position of the Rb^+^ ions has only a small effect on the UV–vis absorptions (Figure S27). For the trianion, the experimental spectrum is better reproduced without Rb^+^, which may indicate that there is no binding or only weak coordination to Rb^+^ in solution. In case of higher reduced **HPH**, the experimental spectra appear to have less structure or are more broadened than the calculated spectra. This could imply that the anions are not always fully coordinated with four Rb^+^ in solution.

The UV–vis spectra of {Rb^+^(18-crown-6)}_4_(**1**^*n*–^) (*n* = 4–6) were also simulated (Figure S28). The presence of the crown ether molecules leads to similar changes as observed for the addition of bare Rb^+^ cations. In fact, the simulated spectra of {Rb^+^(18-crown-6)}_4_(**1**^*n*–^) appear more similar to those of **1**^*n*–^ (without Rb^+^) than to those of Rb^+^_4_(**1**^*n*–^), (Figure S26). The reason is probably that the additional interactions of the Rb^+^ centers with the crown ether ligands in turn weaken the interaction between Rb^+^ and the **HPH** core.

## Conclusions

In summary, the first chemical reduction study of **1** was conducted with alkali metals Na, K, and Rb to reveal its remarkable electron-accepting abilities and to afford a unique family of stepwise-reduced products of **HPH** (**1**) in various reduction states. Notably, in contrast to two-reduction steps revealed electrochemically,^53^ the chemical reduction of **HPH** with alkali metals induces up to 6-fold electron addition. The stepwise-reduced anions of **HPH** were isolated with different countercations as single-crystalline materials, ranging from [**Na-1**^**–**^]·4THF and [**K**_**2**_**-1**^**2–**^]·4C_6_H_14_ to highly reduced [**Rb**_**4**_**-1**^**4–**^]·10THF and [**Rb**_**5**_**-1**^**5–**^]·5THF. The outcomes of one- and two-electron acquisition include the curvature reduction of **HPH** and better π-conjugation over its curved carbon backbone. In contrast, the higher reduction to the tetra- and penta-reduced states leads to a significant geometry change of the **HPH** core from the boat conformation to a recliner-chair shaped structure. In comparison to other curved nanographenes, **HPH** exhibits an advanced electron-accepting ability and can undergo 6-fold reduction vs 4-fold reduction for warped nanographene^48^ and double [7]helicene.^70^ Although the reversible 6-fold reduction was demonstrated by octabenzo[8]circulene,^49^ the revealed boat–chair conformation change of **HPH** upon multielectron addition and metal coordination is unique. Importantly, the charge-dependent conformation change was proven to be reversible by NMR spectroscopy. The computational results indicate that the conformational change from a boat to a chair is driven by the coordination of multiple cationic Rb^+^/crown ether moieties. The revealed remarkable multielectron accepting properties of **HPH** should render its promise for energy storage applications.

## Data Availability

DFT raw data (geometries, excitation energies and intensities) are available on Zenodo https://zenodo.org/doi/10.5281/zenodo.11181723.

## Supporting Information Available

Full experimental details, UV–vis, ^1^H NMR and EPR spectra for all prepared compounds, X-ray crystallographic data for selected products, and computational details (PDF)
