# Supplementary material for: Unveiling the Multielectron Acceptor Properties of π-Expanded Pyracylene: Reversible Boat to Chair Conversion
Source: J Am Chem Soc. 2024 May 14;146(21):14715–23. doi: 10.1021/jacs.4c02314 (PMC11140751; doi:10.1021/jacs.4c02314)
Supplement: Supplementary file 1 — ja4c02314_si_001.pdf [file ja4c02314_si_001.pdf]

Supporting Information: Additional experimental details, materials, and methods

Unveiling the Multi-Electron Acceptor Properties of π-Expanded Pyracylene: Reversible Boat to Chair Conversion

Yikun Zhu[a], Jan Borstelmann[b], Oliver Bertleff[c], John Bergner[b], Zheng Wei[a], Christian Neiss[c], Andreas Görling[c, d], Milan Kivala*[b], Marina A. Petrukhina*[a]

[a] Y. Zhu, Dr. Z. Wei, Prof. Dr. M. A. Petrukhina, Department of Chemistry, University at Albany, State University of New York, Albany, New York 12222, United States

[b] J. Borstelmann, Dr. J. Bergner, Prof. Dr. M. Kivala, Organisch-Chemisches Institut, Universität Heidelberg, Im Neuenheimer Feld 270, 69120 Heidelberg, Germany

[c] O. Bertleff, Dr. C. Neiss, Prof. Dr. A. Görling, Lehrstuhl für Theoretische Chemie, Friedrich-Alexander Universität Erlangen-Nürnberg (FAU), Egerlandstraße 3, 91058 Erlangen, Germany

[d] Prof. Dr. A. Görling, Erlangen National High Performance Computing Center (NHR@FAU), Martensstr. 1, 91058 Erlangen, Germany

Table of Contents

I. Materials and Methods S2

II. UV-vis Spectroscopic Investigation S4

III. EPR Spectroscopic Investigation S8

IV. NMR Spectroscopic Investigation S11

V. Crystal Structure Solution and Refinement S13

VI. Computational Details S22

VII. Computational Results S22

VIII. References S32

I. Materials and Methods

All manipulations were carried out using break-and-seal and glove-box techniques under an atmosphere of argon.[1] Tetrahydrofuran (THF) and hexanes (Sigma Aldrich) were dried over Na/benzophenone and distilled prior to use. Tetrahydrofuran-d_8 (≥99.5 atom %D, Sigma Aldrich) was dried over NaK_2 alloy and vacuum-transferred. Sodium (99.9 %), potassium (98 %), rubidium (99.5 %), 18-crown-6 ether (99 %) and [2.2.2]cryptand (99.0%) were purchased from Sigma Aldrich and used as received. $C_{102}H_{96}$ (**HPH**, **1**) was prepared according to the previously reported procedure.[2] The UV-vis absorption spectra were recorded on a Shimadzu UV-2600i UV-vis spectrophotometer. The ^{1}H NMR spectra were recorded on a Bruker Ascend-500 spectrometer (500 MHz for ^{1}H). Chemical shifts (δ) are reported in parts per million (ppm) and referenced to the resonances of the corresponding solvent used. The low-temperature NMR experiment was controlled by a Cryo Diffusion cryogenic tank probe, and liquid N_2 was used as a cooling source. The EPR spectra were collected with ADANI SPINSCAN X EPR spectrometer with the following parameters: center field = 336.00 mT, sweep width = 15 mT, sweep time = 60 s, modulation amplitude = 100 uT, attenuation = 20 dB, temperature = 30.7 °C. The extreme oxygen- and moisture sensitivity of the reduced products, **Na-1$^{–}$**, **K$_2$-1$^{2–}$**, **Rb$_4$-1$^{4–}$**, and **Rb$_5$-1$^{5–}$**, along with the presence of interstitial hexane and THF molecules, prevented obtaining elemental analysis data.

[Na$^+$(18-crown-6)(THF)$_2$][C$_{102}$H$_{96}$$^–$]·7C$_4H_8$O ([Na-1$^–$]·7C$_4H_8$O)

THF (1.0 mL) was added to a customized glass system containing excess Na metal (3.0 mg, 0.130 mmol), 18-crown-6 (1.0 mg, 0.004 mmol), and **1** (3.0 mg, 0.002 mmol). The reaction mixture was stirred at 25 °C under argon for 20 minutes. The initial purple color (neutral ligand) changed to green in 15 minutes and stayed the same until the reaction was stopped. The mixture was filtered after 20 minutes, and the green filtrate was layered with 1.5 mL of anhydrous hexanes. The ampule was sealed under argon and stored at 5 °C. After 7 days, block-shaped crystals formed in the ampule. Yield: 2.0 mg, 40%. UV-vis (THF): λ_{max} 336, 358, 424, 466, 500, 654, 716, 788 nm.

[K$^+$(cryptand)]$_2$[C$_{102}$H$_{96}$$^{2–}$]·4C$_6H_{14}$ ([K$_2$-1$^{2–}$]·4C$_6$H$_{14}$)

THF (1.0 mL) was added to a customized glass system containing excess K metal (3.0 mg, 0.077 mmol), [2.2.2]cryptand (1.7 mg, 0.004 mmol), and **1** (3.0 mg, 0.002 mmol). The reaction mixture was stirred at 25 °C under argon for 20 minutes. The initial purple color (neutral ligand) changed

to green in 10 minutes, followed by purple in 15 minutes. The mixture was filtered after 20 minutes, and the purple filtrate was layered with 1.5 mL of anhydrous hexanes. The ampule was sealed under argon and stored at 5 °C. After 7 days, block-shaped crystals formed in the ampule. Yield: 2.0 mg, 35%. UV-vis (THF): λ_{max} 365, 550, 764 nm. ^{1}H NMR (THF-d_8, ppm, 25 °C): δ = 1.65 (36H, **1**$^{2-}$), 1.67 (36H, **1**$^{2-}$), 3.99 ([2.2.2]cryptand), 7.87 (8H, **1**$^{2-}$), 8.76 (12H, **1**$^{2-}$), 10.02 (4H, **1**$^{2-}$).

[{Rb^+(18-crown-6)}$_4$($C_{102}H_{96}^{4-}$)]·10C_4H_8O ([Rb_4-1^{4-}]·10C_4H_8O)

THF (1.5 mL) was added to a customized glass system containing excess Rb metal (3.0 mg, 0.035 mmol), 18-crown-6 (1.0 mg, 0.004 mmol) and **1** (3.0 mg, 0.002 mmol). The reaction mixture was stirred at 25 °C under argon for 2 hours. The initial purple color (neutral ligand) changed to green in 10 minutes, followed by purple in 20 minutes and by brownish-purple in 50 minutes. The mixture was filtered after 2 hours, and the dark purple filtrate was layered with 1.0 mL of anhydrous hexanes. The ampule was sealed under argon and stored at 5 °C. After 7 days, block-shaped crystals formed in the ampule. Yield: 2.0 mg, 26%. UV-vis (THF): λ_{max} 484, 587, 744 nm.

[Rb^+(18-crown-6)][{Rb^+(18-crown-6)}$_4$($C_{102}H_{96}^{5-}$)]·5C_4H_8O ([Rb_5-1^{5-}]·5C_4H_8O)

THF (1.5 mL) was added to a customized glass system containing excess Rb metal (3.0 mg, 0.035 mmol), 18-crown-6 (1.0 mg, 0.004 mmol), and **1** (3.0 mg, 0.002 mmol). The reaction mixture was stirred at 25 °C under argon for 24 hours. The initial purple color (neutral ligand) changed to green in 10 minutes, followed by purple in 20 minutes and by brownish-purple in 50 minutes. The mixture was filtered after 24 hours, and the dark purple filtrate was layered with 1.0 mL of anhydrous hexanes. The ampule was sealed under argon and stored at 5 °C. After 7 days, block-shaped crystals formed in the ampule. Yield: 2.0 mg, 26%. UV-vis (THF): λ_{max} 353, 485, 742 nm.

II. UV-vis Spectroscopic Investigation

Sample preparation: THF (3 mL) was added to a quartz cuvette containing **1** (0.2 mg, 0.0001 mmol), 18-crown-6 (0.1 mg, 0.0004 mmol), and Na metal (1.0 mg, 0.043 mmol). The ampule was sealed with Teflon stopper under argon, and UV-vis absorption spectra were monitored at different reaction times at 25 °C.

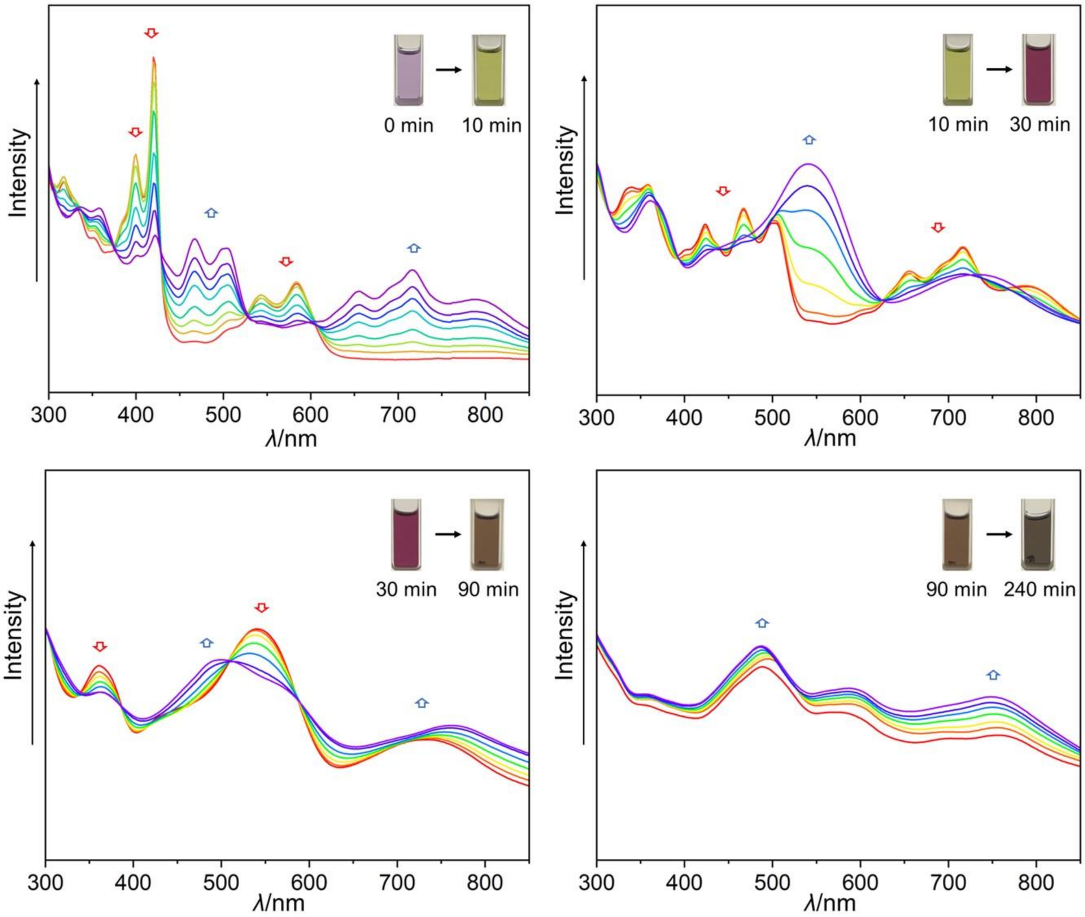

Figure S1. UV-vis spectra of Na/**1**/18-crown-6 in THF.

Sample preparation: THF (3 mL) was added to a quartz cuvette containing **1** (0.2 mg, 0.0001 mmol), [2.2.2]cryptand (0.1 mg, 0.0003 mmol), and K metal (1.0 mg, 0.026 mmol). The ampule was sealed with Teflon stopper under argon, and UV-vis absorption spectra were monitored at different reaction times at 25 °C.

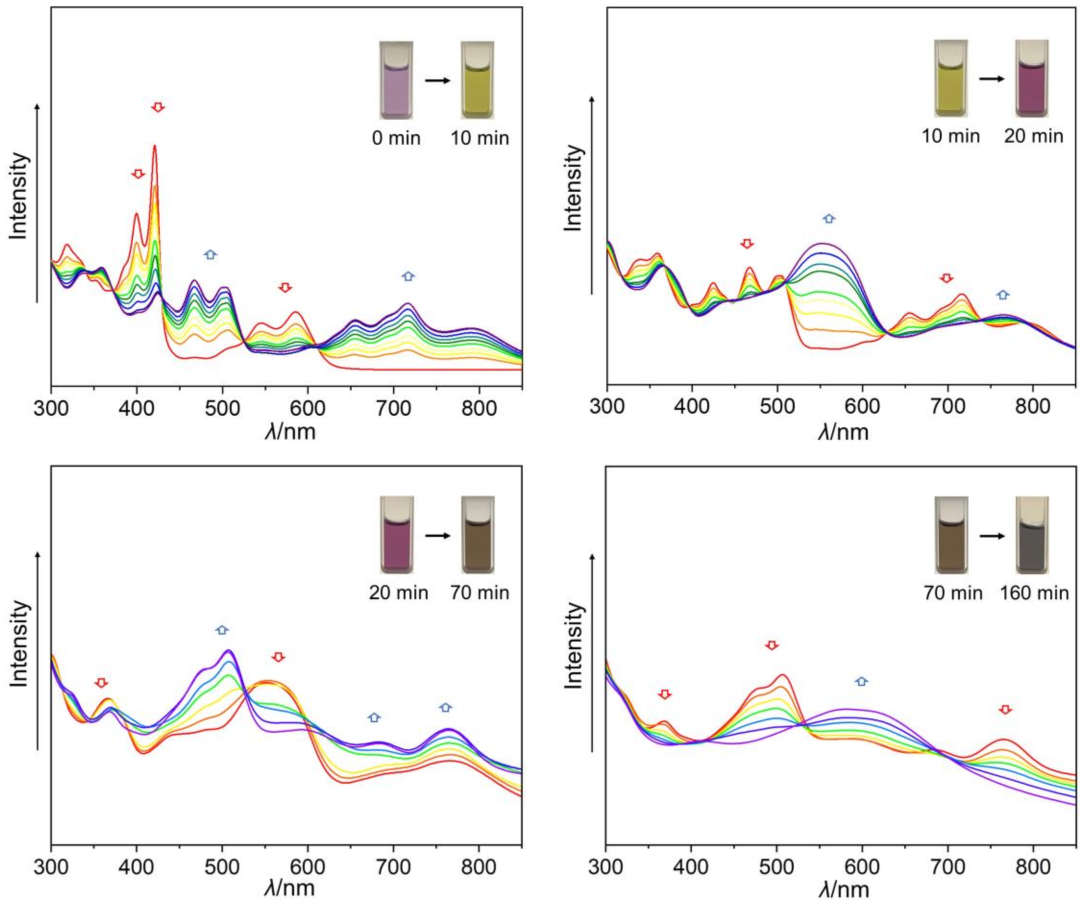

Figure S2. UV-vis spectra of K/**1**/[2.2.2]cryptand in THF.

Sample preparation: THF (3 mL) was added to a quartz cuvette containing **1** (0.2 mg, 0.0001 mmol), 18-crown-6 (0.2 mg, 0.0007 mmol), and Rb metal (1.0 mg, 0.0117 mmol). The ampule was sealed with Teflon stopper under argon, and UV-vis absorption spectra were monitored at different reaction times at 25 °C.

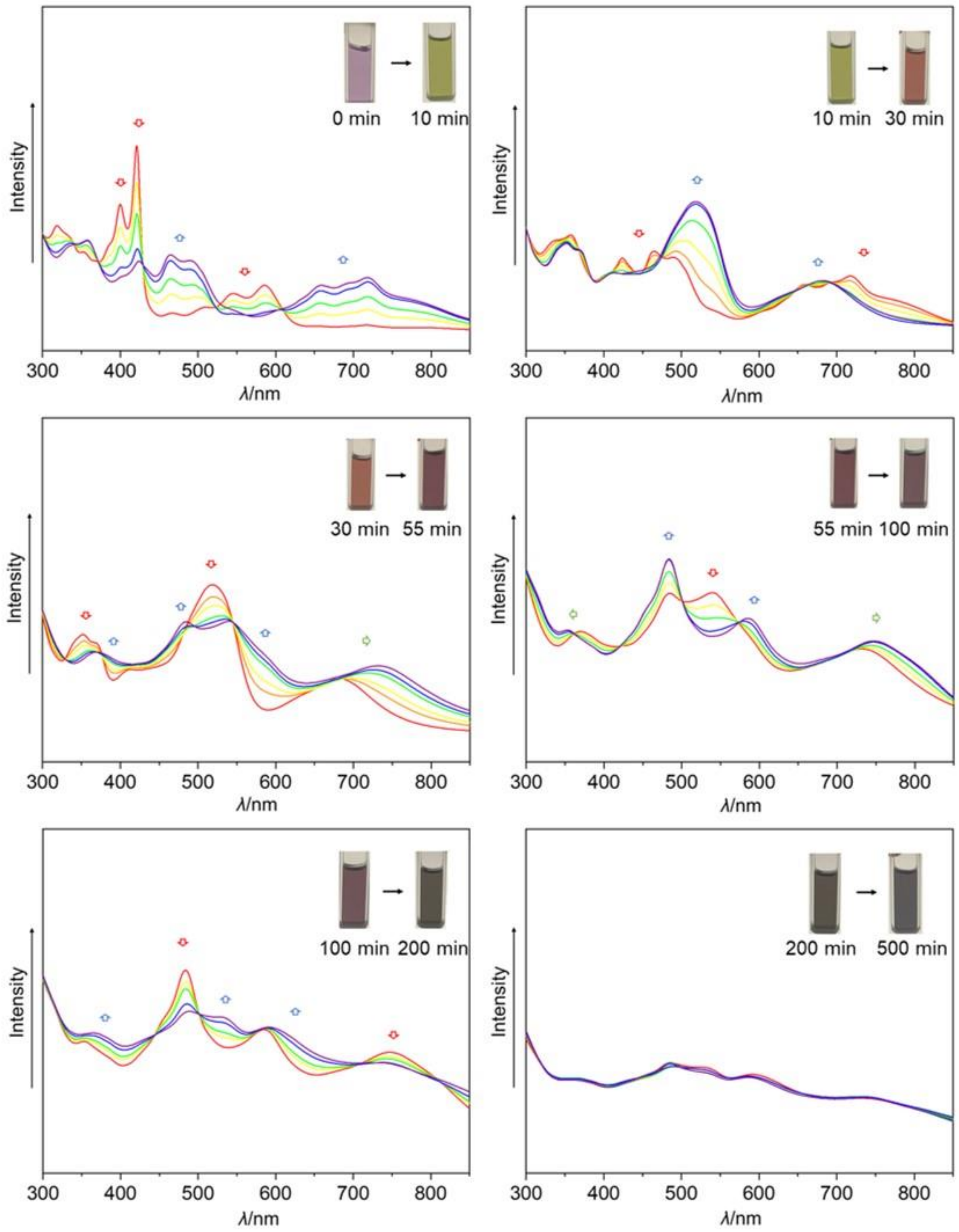

Figure S3. UV-vis spectra of Rb/**1**/18-crown-6 in THF.

Sample preparation: THF (3 mL) was added to a quartz cuvette containing **1** (0.2 mg, 0.0001 mmol) and Rb metal (1.0 mg, 0.0117 mmol). The ampule was sealed with Teflon stopper under argon, and UV-vis absorption spectra were monitored at different reaction times at 25 °C.

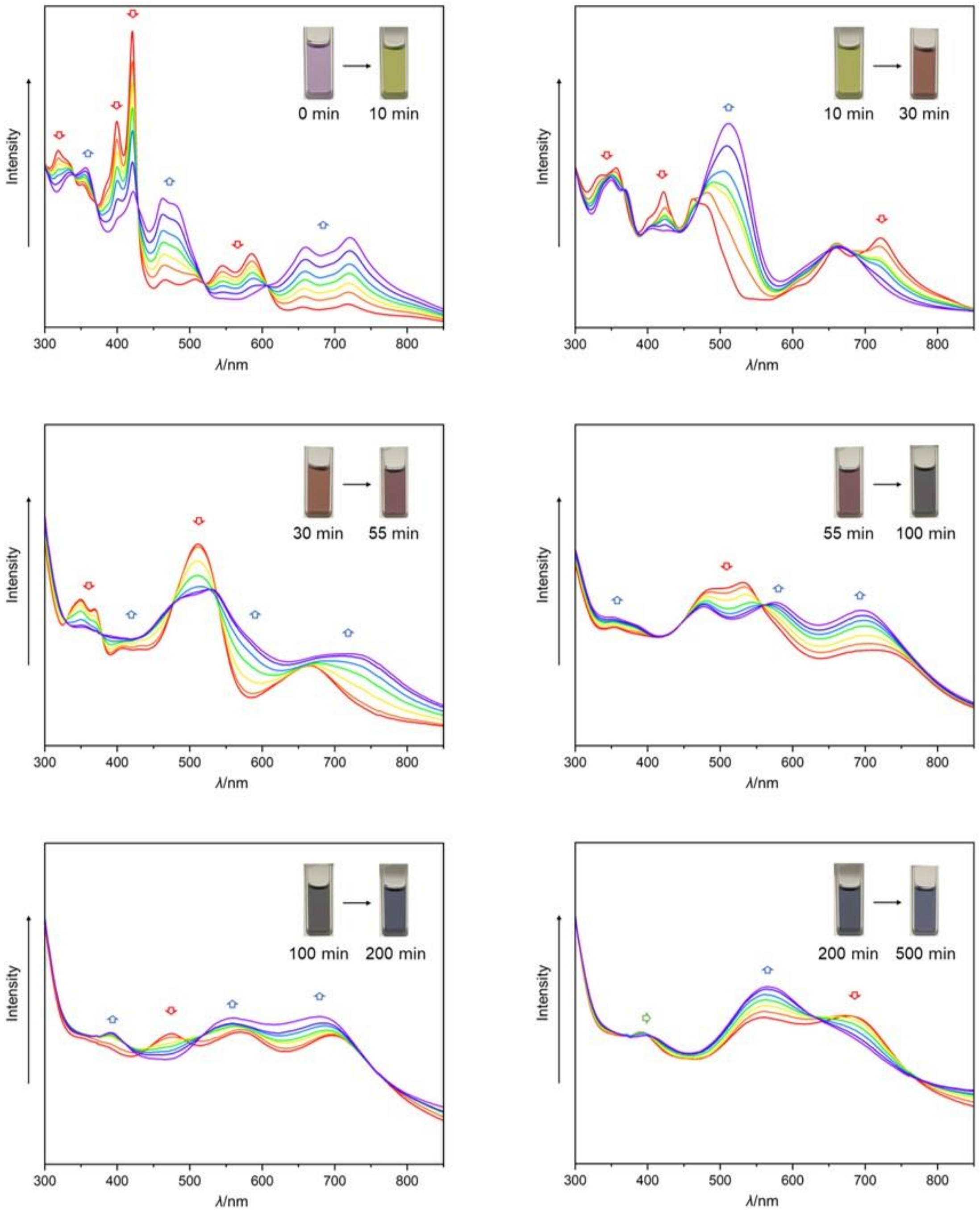

Figure S4. UV-vis spectra of Rb/**1** in THF.

III. EPR Spectroscopic Investigation

Sample preparation: Crystalline sample of **Na-1**$^{-}$ (~1.0 mg) was washed with fresh hexanes, dried under vacuum, grinded and transferred into a glass tube (O. D. 5 mm). The tube was sealed under argon, and the EPR spectrum was collected at 30 °C.

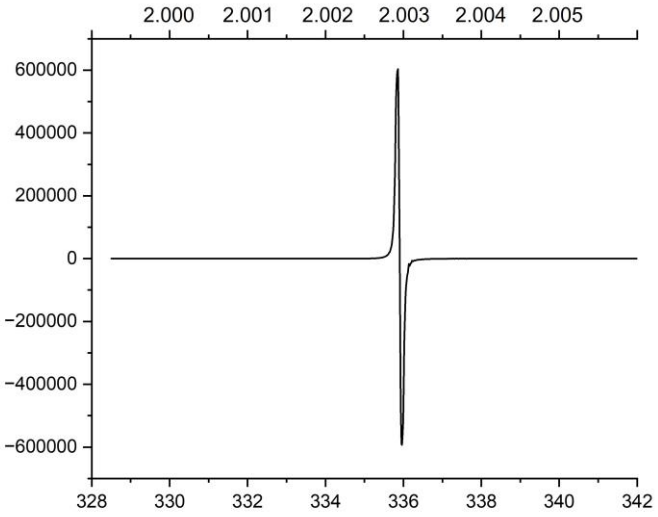

Figure S5. EPR spectrum of a crystalline sample of **Na-1**$^{-}$ at 30 °C, g = 2.0027.

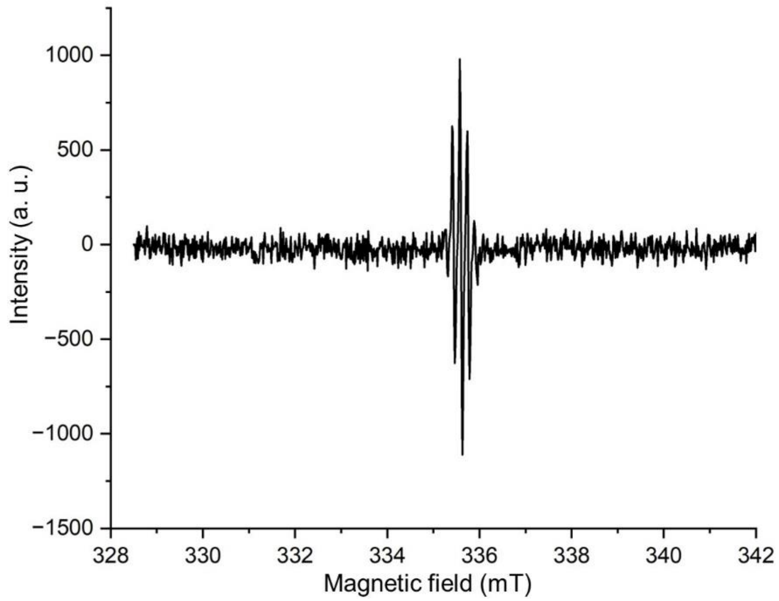

Figure S6. EPR spectrum of crystals of **Na-1**$^{-}$ dissolved in THF at 30 °C, g = 2.0027.

Sample preparation: Crystalline sample of **Rb_5-1^{5-}** (~1.0 mg) was washed with fresh hexanes, dried under vacuum, grinded and transferred into a glass tube (O. D. 5 mm). The tube was sealed under argon, and the EPR spectrum was collected at 30 °C.

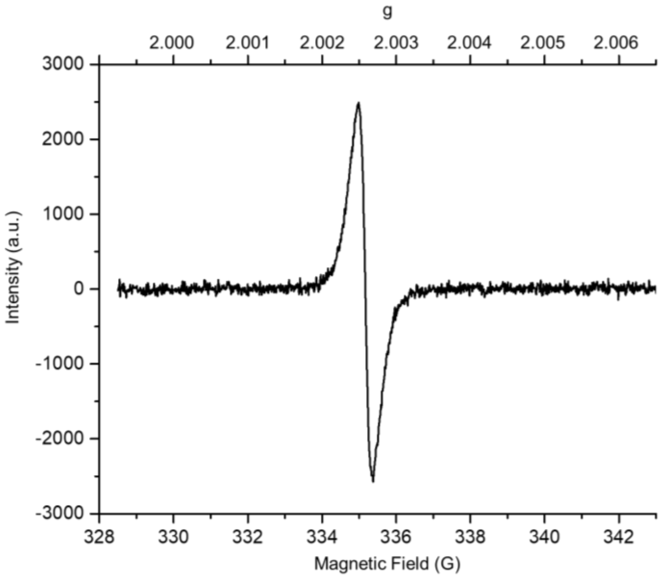

Figure S7. EPR spectrum of a crystalline sample of **Rb-1^{5-}** at 30 °C, g = 2.0024.

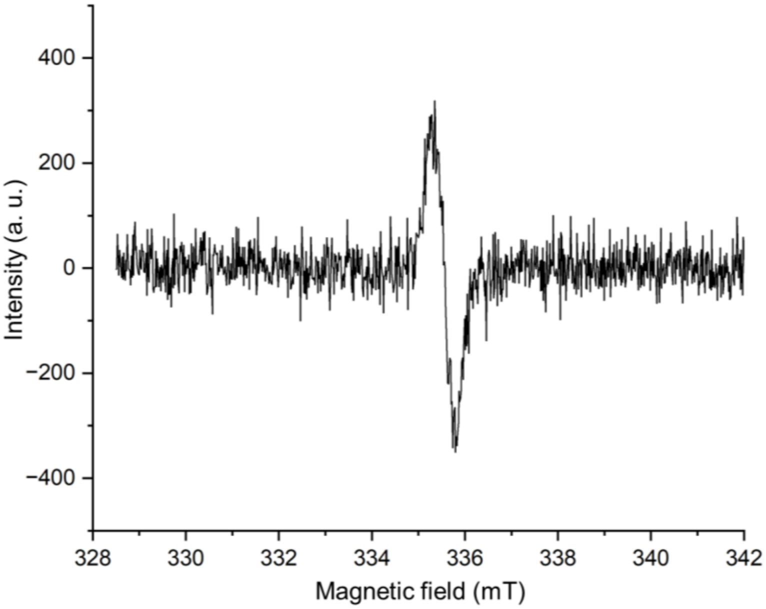

Figure S8. EPR spectrum of crystals of **Rb_5-1^{5-}** dissolved in THF at 30 °C, g = 2.0024.

Sample preparation: THF (0.05 mL) was added to a glass tube (O. D. 5 mm) containing excess Rb metal (3.0 mg, 0.035 mmol) and **1** (1.0 mg, 0.0007 mmol). The tube was sealed under argon and EPR spectra were monitored at different reaction times at 25 °C.

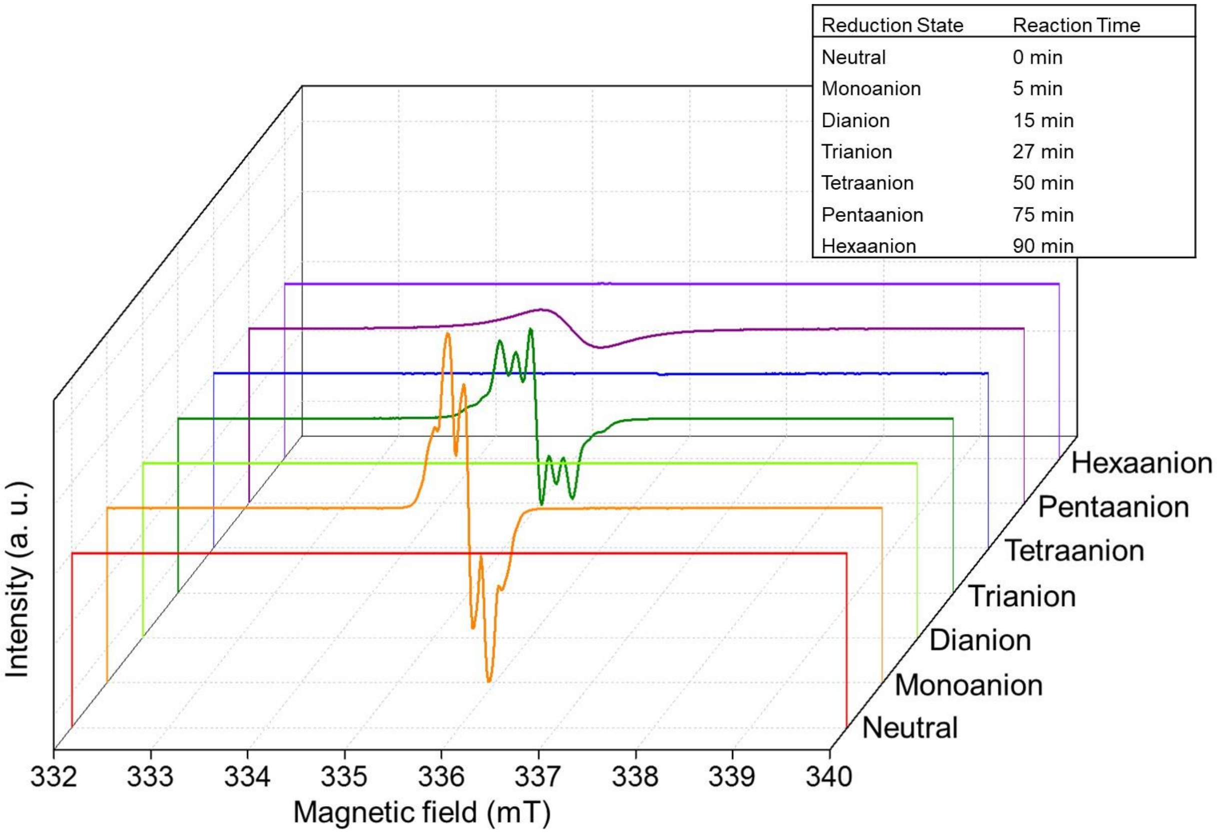

Figure S9. EPR spectra of *in situ* generated anions of **1** at 30 °C.

IV. NMR Spectroscopic Investigation

Sample preparation: THF-d_8 (0.60 mL) was added to an NMR tube containing excess K metal (2.0 mg, 0.051 mmol), [2.2.2]cryptand (1.2 mg, 0.0032 mmol), and **1** (2.0 mg, 0.0015 mmol). The tube was sealed under argon. The ^{1}H NMR spectra of **1** were collected immediately, and that of *in situ* generated $\mathbf{1}^{2-}$ were collected after 15 minutes.

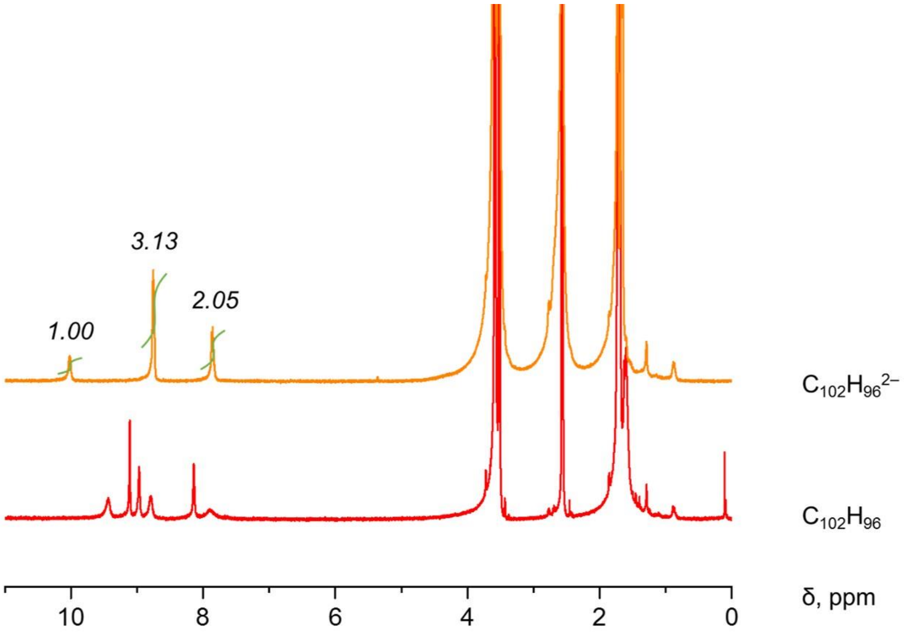

Figure S10. ^{1}H NMR spectra of **1** and *in situ* generated $\mathbf{1}^{2-}$ at 25 °C in THF-d_8.

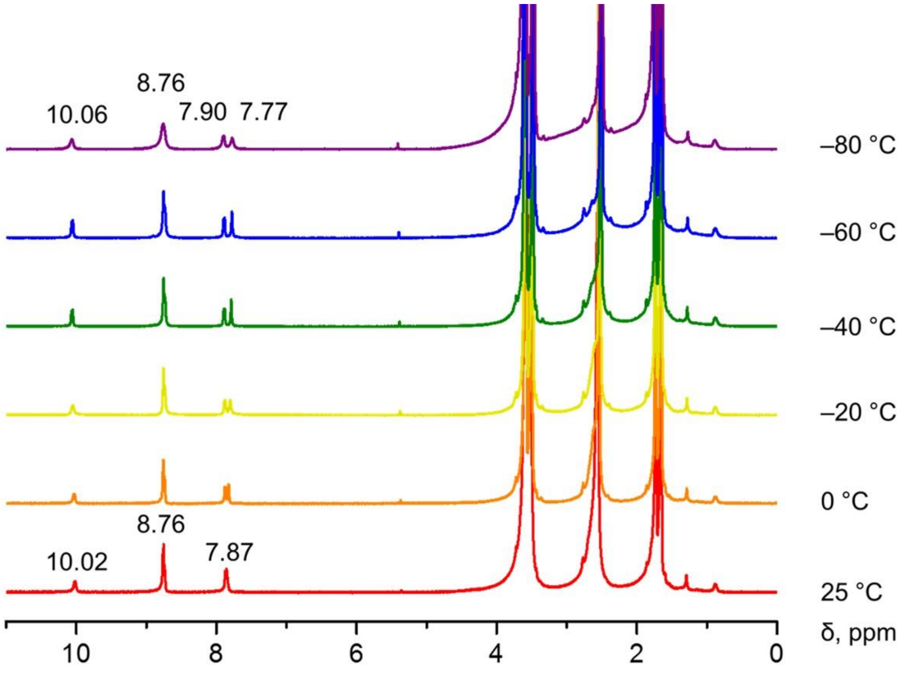

Figure S11. Variable temperature ^{1}H NMR spectra of *in situ* generated $\mathbf{1}^{2-}$ in THF-d_8.

Sample preparation: THF-d_8 (0.70 mL) was added to an NMR tube containing excess Rb metal (3.0 mg, 0.035 mmol) and **1** (2.0 mg, 0.0015 mmol). The tube was sealed under argon, and ^{1}H NMR spectra were monitored at different reaction times at 25 °C.

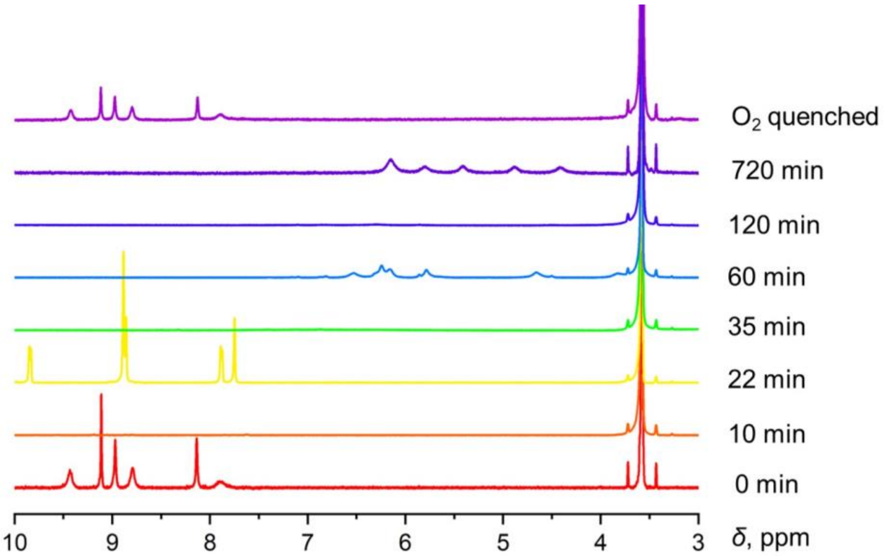

Figure S12. ^{1}H NMR spectra of *in situ* generated anions of **1**, along with the product quenched by O_2, at 25 °C in THF-d_8, aromatic region.

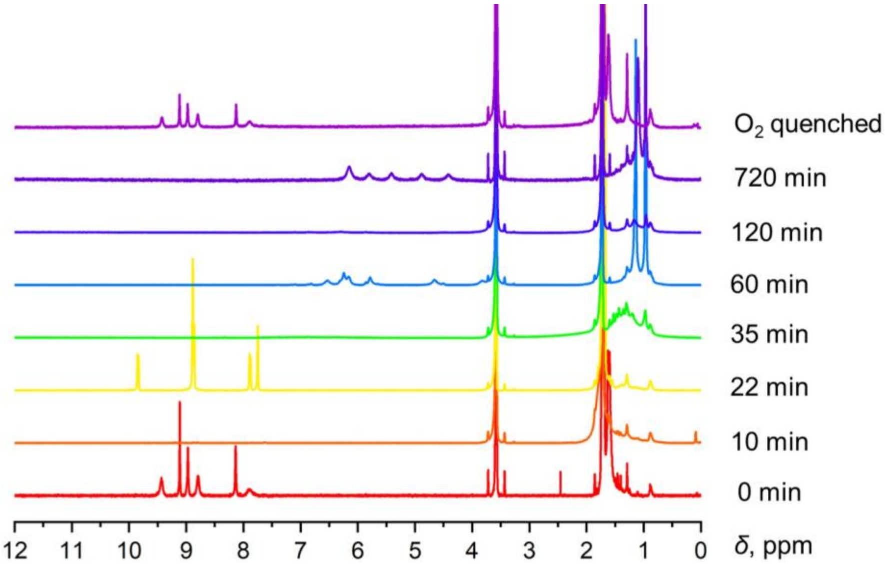

Figure S13. ^{1}H NMR spectra of *in situ* generated anions of **1**, along with the product quenched by O_2, at 25 °C in THF-d_8, full spectra.

V. Crystal Structure Solution and Refinement

Data collections of **Na-1**$^{-}$, **K$_2$-1**$^{2-}$, and **Rb$_5$-1**$^{5-}$ were performed at 100(2) K on a Huber Kappa 4-Circle diffraction system with a DECTRIS PILATUS3 X 2M(CdTe) pixel array detector using ϕ scans (synchrotron radiation at λ = 0.41328 Å) located at the Advanced Photon Source, Argonne National Laboratory (NSF's ChemMatCARS, Sector 15, Beamline 15-ID-D). The dataset's reduction and integration were performed with the Bruker software package SAINT (version 8.38A).[3] Data were corrected for absorption effects using the empirical methods as implemented in SADABS (version 2016/2).[4] Data collection of **Rb$_4$-1**$^{4-}$ was performed at 100.00(10) K on a Rigaku XtaLAB Synergy-S X-ray diffractometer equipped with a HyPix-6000HE hybrid photon counting (HPC) detector and microfocus Cu-Kα radiation (λ = 1.54178 Å). Data collection strategy to ensure completeness and desired redundancy were determined using CrysAlis^{Pro}.[5] Data processing was performed using CrysAlis^{Pro}.[5] Empirical absorption correction was applied using the SCALE3 ABSPACK scaling algorithm.[6] All structures were solved by SHELXT (version 2018/2)[7] and refined by full-matrix least-squares procedures using the Bruker SHELXTL (version 2019/2 or 2019/3)[8] software package through the OLEX2 graphical interface.[9] All non-hydrogen atoms, including those in disordered parts, were refined anisotropically. Hydrogen atoms were included in idealized positions for structure factor calculations with U_{iso}(H) = 1.2 U_{eq}(C) and U_{iso}(H) = 1.5 U_{eq}(C) for methyl groups. In the structure of **Na-1**$^{-}$, five *tert*-butyl groups, two THF and one 18-crown-6 molecules were found to be disordered. In the structure of **K$_2$-1**$^{2-}$, seven *tert*-butyl groups were found to be disordered. In the structure of **Rb$_5$-1**$^{5-}$, two *tert*-butyl groups and one and half 18-crown-6 molecules were found to be disordered. In the structure of **Rb$_4$-1**$^{4-}$, two *tert*-butyl groups, four THF molecules, and one rubidium cation were found to be disordered. All disordered molecules and ions were modeled with two orientations with their relative occupancies refined. The geometries of the disordered parts were restrained to be similar. The anisotropic displacement parameters of all the disordered groups and molecules were restrained to have the same U_{ij} components, with a standard uncertainty of 0.01 Å^2. In each unit cell of **Na-1**$^{-}$, twenty eight THF solvent molecules were found to be severely disordered and removed by the Olex2's solvent mask subroutine.[9] The total void volume was 3996.6 Å^3, equivalent to 30.95 % of the unit cell's total volume. In each unit cell of **K$_2$-1**$^{2-}$, eight THF solvent molecules were found to be severely disordered and removed by the Olex2's solvent mask subroutine.[9] The total void volume was 1994.3 Å^3, equivalent to 27.46 % of the unit cell's total volume. In each unit cell of **Rb$_5$-1**$^{5-}$,

three THF solvent molecules were found to be severely disordered and removed by the Olex2's solvent mask subroutine.[9] The total void volume was 547.3 Å^3, equivalent to 13.16 % of the unit cell's total volume. Further crystal and data collection details are listed in Table S1.

Table S1. Crystallographic data of **Na-1$^–$**, **K$_2$-1$^{2–}$**, **Rb$_4$-1$^{4–}$**, and **Rb$_5$-1$^{5–}$**.

Compound	**Na-1$^–$**	**K$_2$-1$^{2–}$**	**Rb$_4$-1$^{4–}$**	**Rb$_5$-1$^{5–}$**
Empirical formula	$C_{150}H_{192}NaO_{15}$	$C_{162}H_{224}K_2O_{12}N_4$	$C_{190}H_{272}Rb_4O_{34}$	$C_{174}H_{240}Rb_5O_{33}$
Formula weight	2258.01	2497.64	3441.94	3287.00
Temperature (K)	100(2)	100(2)	100.00(10)	100(2)
Wavelength (Å)	0.41328	0.41328	1.54178	0.41328
Crystal system	Triclinic	Triclinic	Triclinic	Triclinic
Space group	*P*-1	*P*-1	*P*-1	*P*-1
a (Å)	18.732(2)	17.780(2)	12.3562(2)	14.1444(13)
b (Å)	24.355(3)	18.909(2)	16.6280(3)	14.3807(14)
c (Å)	29.160(4)	23.545(3)	21.9340(4)	21.513(2)
α (°)	85.740(3)	98.028(2)	86.518(2)	74.663(2)
β (°)	88.508(4)	104.089(2)	83.819(2)	81.237(2)
γ (°)	76.738(3)	104.483(2)	83.595(3)	83.515(2)
V (Å^3)	12912(3)	7262.3(15)	4446.96(14)	4158.6(7)
Z	4	2	1	1
ρ_{calcd} (g·cm^{-3})	1.162	1.142	1.285	1.313
μ (mm^{-1})	0.033	0.043	1.952	0.377
F(000)	4892	2716	1832	1733
Crystal size (mm)	0.03×0.05×0.06	0.008×0.008×0.011	0.008×0.008×0.011	0.005×0.009×0.011
θ range for data collection (°)	0.724-14.230	1.010-14.245	2.677-79.478	1.085-14.987
Reflections collected	136179	157256	132036	104703
Independent reflections	42563 [R_{int} = 0.2024]	23931 [R_{int} = 0.0668]	66296 [R_{int} = 0.0381]	15926 [R_{int} = 0.0585]
Transmission factors (min/max)	0.6303/0.7438	0.5689/0.7438	0.8680/1.0000	0.5431/0.6515
Data/restraints/params.	42563/1372/2803	23931/666/1670	66296/726/1266	15926/1024/1208
$R1$,[a] $wR2$[b] ($I > 2\sigma(I)$)	0.0941, 0.2376	0.1005, 0.2948	0.0544, 0.1417	0.0538, 0.1448
$R1$,[a] $wR2$[b] (all data)	0.1843, 0.2901	0.1200, 0.3119	0.0666, 0.1498	0.0585, 0.1477
Quality-of-fit[c]	0.932	1.046	1.038	1.051

$R_{int} = \Sigma|F_o^2 - \langle F_o^2 \rangle| / \Sigma|F_o^2|$

[a]$R1 = \Sigma||F_o|-|F_c||/\Sigma|F_o|$. [b]$wR2 = [\Sigma[w(F_o^2-F_c^2)^2]/\Sigma[w(F_o^2)^2]]$.

[c]Quality-of-fit = $[\Sigma[w(F_o^2-F_c^2)^2]/(N_{obs}-N_{params})]^{½}$, based on all data.

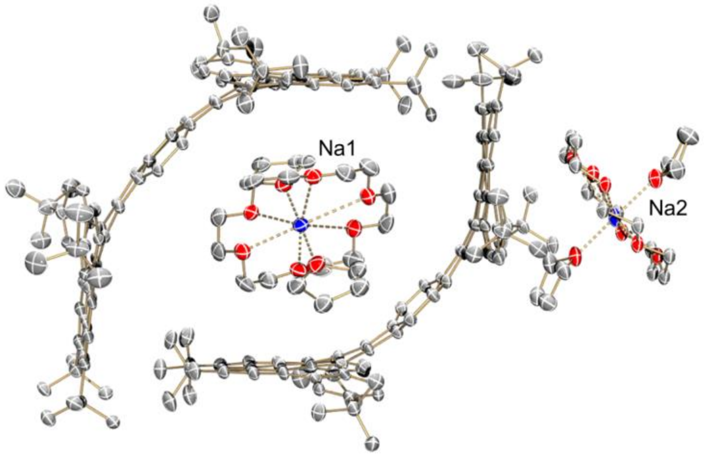

Figure S14. ORTEP drawing of the asymmetric unit of **Na-1**$^{-}$ with thermal ellipsoids at the 30% probability level. Hydrogen atoms are omitted for clarity. Color key: C gray, O red, Na blue.

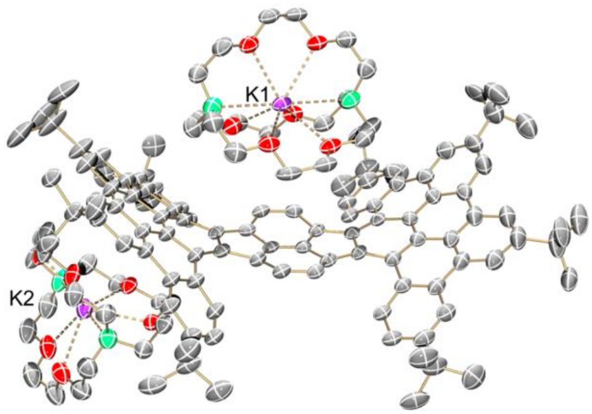

Figure S15. ORTEP drawing of the asymmetric unit of **K$_2$-1**$^{2-}$ with thermal ellipsoids at the 40% probability level. Hydrogen atoms are omitted for clarity. Color key: C gray, O red, N spring green, K dark orchid.

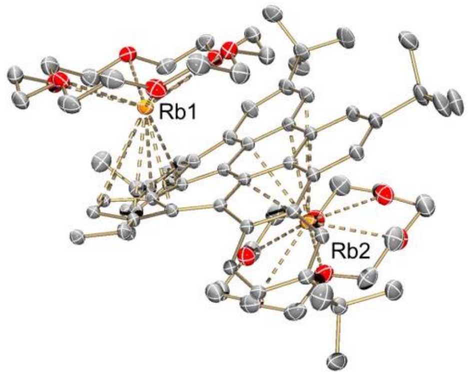

Figure S16. ORTEP drawing of the asymmetric unit of **Rb$_4$-1**$^{4-}$ with thermal ellipsoids at the 40% probability level. Due to symmetry, only half of the molecule is shown in the asymmetric unit. Hydrogen atoms and interstitial solvent molecules are omitted for clarity. Color key: C gray, O red, Rb orange.

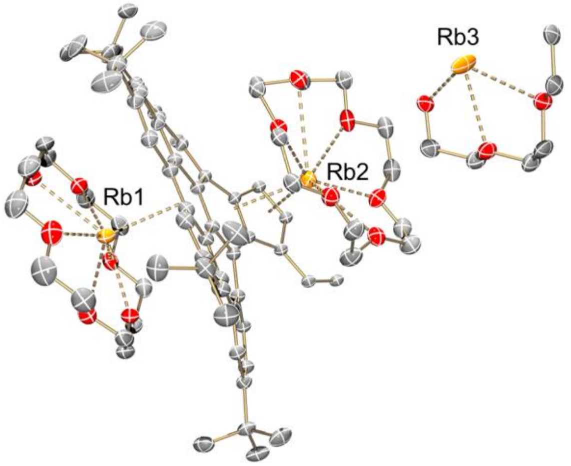

Figure S17. ORTEP drawing of the asymmetric unit of **Rb_5-1^{5-}** with thermal ellipsoids at the 40% probability level. Due to symmetry, only half of the molecule is shown in the asymmetric unit. Hydrogen atoms are omitted for clarity. Color key: C gray, O red, Rb orange.

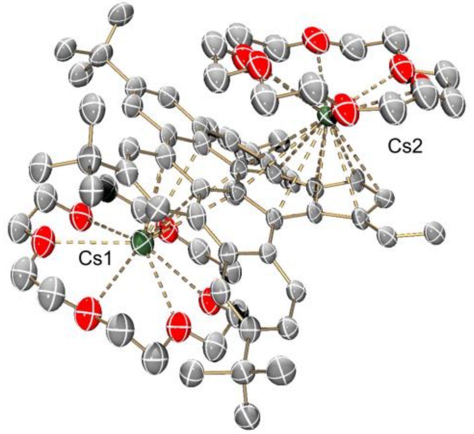

Figure S18. ORTEP drawing of the asymmetric unit of **Cs_4-1^{4-}** with thermal ellipsoids at the 30% probability level. Due to symmetry, only half of the molecule is shown in the asymmetric unit. Hydrogen atoms and interstitial solvent molecules are omitted for clarity. Color key: C gray, O red, Cs dark green.

Note: The diffraction of this crystal was very weak and only reached a 2-theta value of 95 degrees (a resolution of 1.05 Å). However, this data set has a data-to-parameter ratio up to ~12 and the structure completeness of 0.991, indicating that the identity and atomic connectivity of the structure model derived from these data are reliable. The model confirms the chair-conformation of the **HPH** tetra-anion and clearly identifies the alkali metals binding sites (see Figure S17). Unfortunately, the overall poor data quality prevents any further structural analysis (bond lengths/angles) and comparison. Multiple crystals from the Cs-induced reduction reactions of **HPH** were checked and none has provided better quality data.

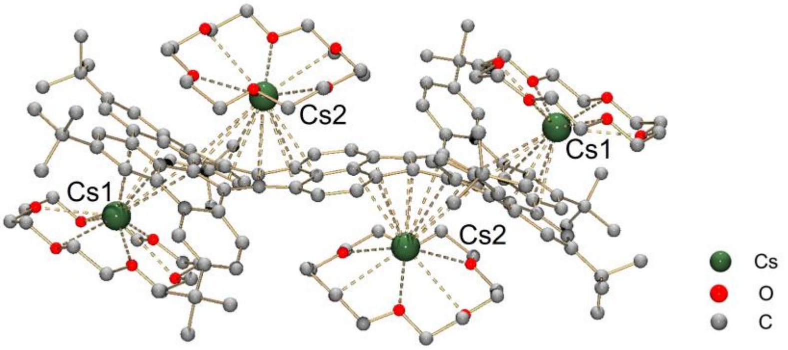

Figure S19. Crystal structure of $\mathbf{Cs_4\text{-}1^{4-}}$, ball-and-stick model. Hydrogen atoms are omitted for clarity.

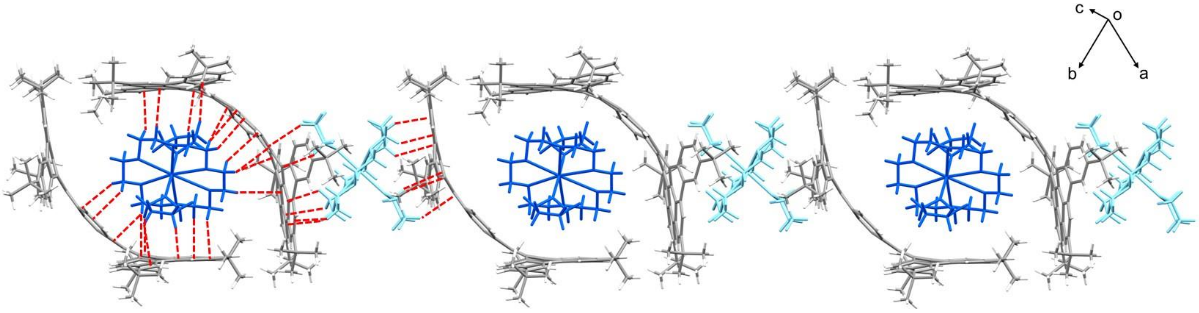

Figure S20. 1D column packing in $\mathbf{Na\text{-}1^{-}}$, capped stick model. C–H$\cdots\pi$ interactions (2.584(7)–3.010(7) Å) between the cationic moieties and the $\mathbf{1}^{-}$ cores are shown in red. Independent cations are highlighted in different shades of blue.

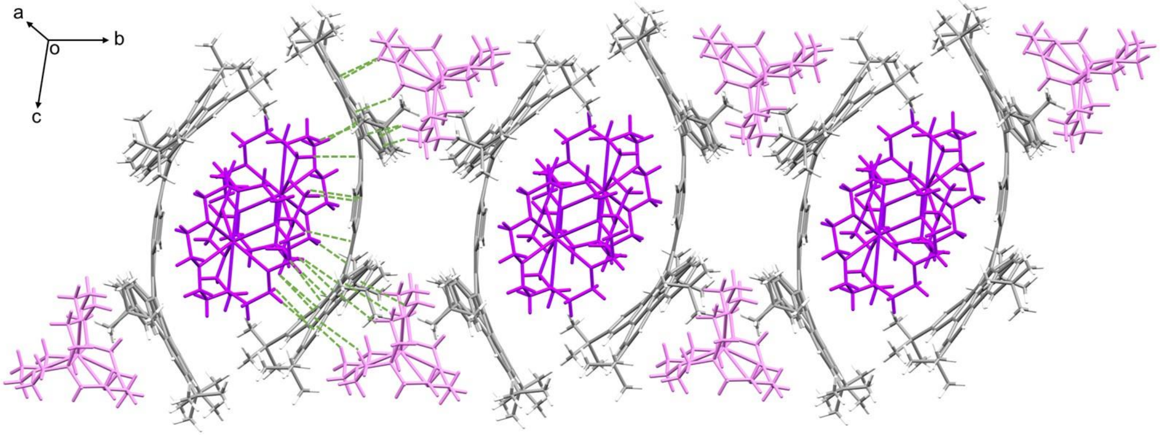

Figure S21. 1D column packing in $\mathbf{K_2\text{-}1^{2-}}$, capped stick model. C–H$\cdots\pi$ interactions (2.362(7)–3.041(7) Å) between the cationic moieties and the $\mathbf{1}^{-}$ cores are shown in green. Independent cations are highlighted in different shades of purple.

Table S2. Rb–C distances (Å) in **Rb_4-1^{4-}** (left) and **Rb_5-1^{5-}** (right), along with a labeling scheme.

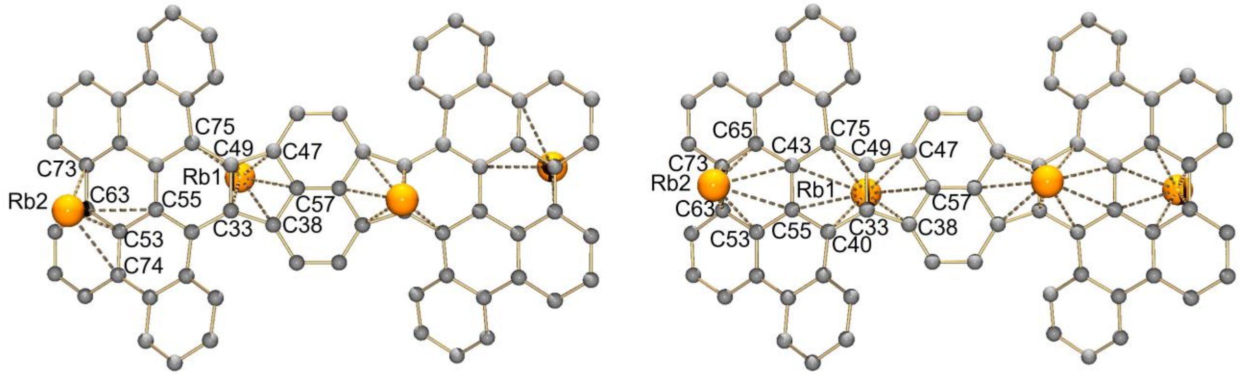

	**Rb_4-1^{4-}**	**Rb_5-1^{5-}**		**Rb_4-1^{4-}**	**Rb_5-1^{5-}**
Rb1–C33	3.273(2)	3.121(2)	Rb2–C53	3.140(2)	3.271(2)
Rb1–C38	3.487(2)	3.421(2)	Rb2–C55	3.482(2)	3.380(2)
Rb1–C47	3.276(2)	3.366(3)	Rb2–C63	3.246(3)	3.299(2)
Rb1–C49	3.136(2)	3.071(2)	Rb2–C73	3.542(3)	3.376(2)
Rb1–C57	3.423(2)	3.495(2)	Rb2–C74	3.646(3)	-
Rb1–C75	3.587(2)	3.428(1)	Rb2–C43	-	3.411(2)
Rb1–C43	-	3.520(2)	Rb2–C65	-	3.372(2)
Rb1–C55	-	3.598(2)			
Rb1–C40	-	3.561(2)			

Table S3. C–C bond distances (Å) in **1**, **1**$^{-}$ and **1**$^{2-}$, along with a labeling scheme.

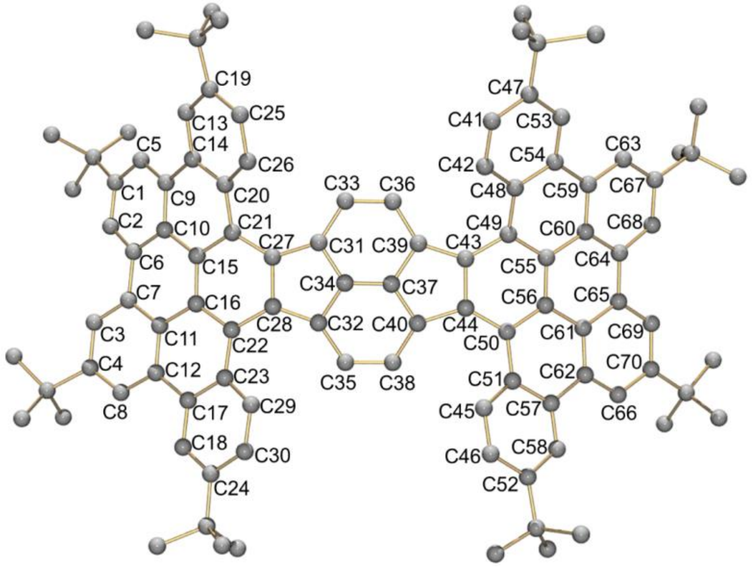

	**1**	**1**$^{-}$	**1**$^{2-}$		**1**	**1**$^{-}$	**1**$^{2-}$
C1–C2	1.378	1.382(6)	1.357(7)	C35–C38	1.429	1.386(6)	1.383(7)
C1–C5	1.378	1.394(6)	1.367(7)	C36–C39	1.385	1.406(6)	1.428(7)
C2–C6	1.404	1.387(6)	1.408(7)	C37–C39	1.406	1.422(6)	1.399(7)
C3–C4	1.393	1.382(6)	1.380(7)	C37–C40	1.399	1.414(6)	1.442(7)
C3–C7	1.406	1.397(6)	1.393(7)	C38–C40	1.385	1.418(6)	1.420(7)
C4–C8	1.385	1.397(6)	1.373(7)	C39–C43	1.500	1.463(6)	1.447(7)
C5–C9	1.390	1.393(6)	1.396(7)	C40–C44	1.496	1.467(6)	1.421(7)
C6–C7	1.453	1.458(6)	1.447(7)	C41–C42	1.376	1.358(6)	1.375(7)
C6–C10	1.419	1.418(6)	1.409(7)	C41–C47	1.401	1.417(6)	1.385(7)
C7–C11	1.410	1.423(6)	1.420(7)	C42–C48	1.391	1.426(6)	1.404(7)
C8–C12	1.396	1.394(6)	1.385(7)	C43–C44	1.401	1.427(6)	1.448(7)
C9–C10	1.411	1.407(6)	1.406(7)	C43–C49	1.419	1.426(6)	1.407(7)
C9–C14	1.479	1.488(6)	1.453(7)	C44–C50	1.412	1.411(6)	1.435(7)
C10–C15	1.440	1.460(6)	1.437(7)	C45–C46	1.370	1.369(6)	1.365(7)
C11–C12	1.419	1.409(6)	1.390(7)	C45–C51	1.401	1.416(6)	1.390(7)
C11–C16	1.442	1.441(6)	1.432(7)	C46–C52	1.395	1.401(6)	1.385(7)
C12–C17	1.482	1.478(6)	1.488(7)	C47–C53	1.379	1.383(6)	1.376(7)
C13–C14	1.402	1.396(6)	1.384(7)	C48–C49	1.454	1.445(6)	1.451(7)
C13–C19	1.360	1.400(6)	1.371(7)	C48–C54	1.415	1.406(6)	1.416(7)
C14–C20	1.403	1.404(6)	1.426(7)	C49–C55	1.415	1.420(6)	1.416(7)
C15–C16	1.406	1.422(6)	1.406(7)	C50–C51	1.462	1.456(6)	1.432(7)
C15–C21	1.425	1.408(6)	1.410(7)	C50–C56	1.429	1.430(6)	1.409(7)
C16–C22	1.424	1.416(6)	1.413(7)	C51–C57	1.413	1.401(6)	1.421(7)
C17–C18	1.393	1.408(6)	1.355(7)	C52–C58	1.373	1.387(6)	1.368(7)
C17–C23	1.411	1.404(6)	1.415(7)	C53–C54	1.395	1.398(6)	1.403(7)
C18–C24	1.380	1.373(6)	1.400(7)	C54–C59	1.469	1.464(6)	1.438(7)
C19–C25	1.399	1.391(6)	1.387(7)	C55–C56	1.406	1.415(6)	1.419(7)
C20–C21	1.457	1.460(6)	1.443(7)	C55–C60	1.437	1.439(6)	1.430(7)

C20–C26	1.413	1.405(6)	1.389(7)	C56–C61	1.449	1.447(6)	1.417(7)
C21–C27	1.412	1.411(6)	1.429(7)	C57–C58	1.400	1.398(6)	1.383(7)
C22–C23	1.448	1.446(6)	1.447(7)	C57–C62	1.469	1.476(6)	1.472(7)
C22–C28	1.406	1.422(6)	1.406(7)	C59–C60	1.416	1.420(6)	1.402(7)
C23–C29	1.404	1.419(6)	1.404(7)	C59–C63	1.383	1.399(6)	1.401(7)
C24–C30	1.402	1.399(6)	1.396(7)	C60–C64	1.418	1.414(6)	1.403(7)
C25–C26	1.367	1.373(6)	1.364(7)	C61–C62	1.402	1.409(6)	1.407(7)
C27–C28	1.414	1.446(6)	1.440(7)	C61–C65	1.421	1.418(6)	1.412(7)
C29–C30	1.367	1.365(6)	1.341(7)	C62–C66	1.393	1.386(6)	1.402(7)
C27–C31	1.494	1.457(6)	1.436(7)	C63–C67	1.374	1.384(6)	1.379(7)
C28–C32	1.493	1.471(6)	1.462(7)	C64–C65	1.456	1.475(6)	1.461(7)
C31–C33	1.370	1.412(6)	1.426(7)	C64–C68	1.393	1.402(6)	1.405(7)
C31–C34	1.401	1.410(6)	1.414(7)	C65–C69	1.390	1.399(6)	1.383(7)
C32–C34	1.401	1.392(6)	1.383(7)	C66–C70	1.381	1.381(6)	1.361(7)
C32–C35	1.376	1.407(6)	1.402(7)	C67–C68	1.389	1.395(6)	1.339(7)
C33–C36	1.434	1.398(6)	1.380(7)	C69–C70	1.385	1.395(6)	1.368(7)
C34–C37	1.381	1.373(6)	1.350(7)				

* The C–C bonds were shown in red/blue when the distances are shorter/longer compared to the distances in **1**.

Table S4. C–C bond distances (Å) in $\mathbf{1}^{4-}$ and $\mathbf{1}^{5-}$, along with a labeling scheme.

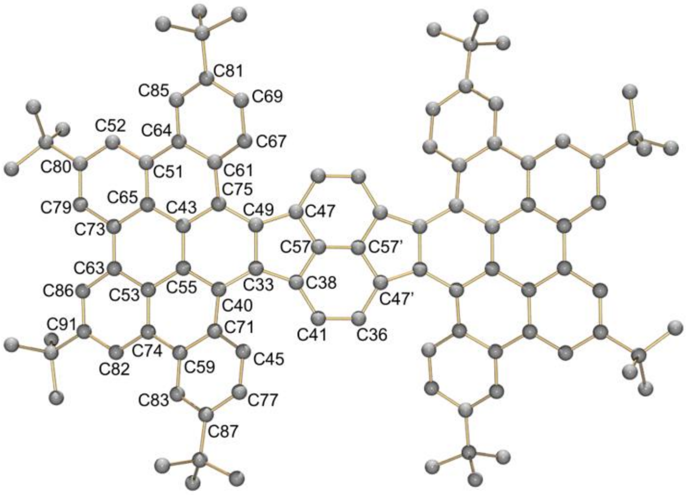

	$\mathbf{1}^{4-}$	$\mathbf{1}^{5-}$		$\mathbf{1}^{4-}$	$\mathbf{1}^{5-}$
C36–C47’	1.440(4)	1.426(3)	C51–C52	1.432(4)	1.428(3)
C47–C57	1.416(4)	1.415(3)	C52–C80	1.404(4)	1.394(4)
C57–C57’	1.385(4)	1.370(4)	C79–C80	1.393(4)	1.379(4)
C38–C57	1.416(4)	1.402(3)	C73–C79	1.436(4)	1.425(3)
C38–C41	1.448(4)	1.440(3)	C65–C73	1.427(4)	1.425(3)
C36–C41	1.385(4)	1.383(3)	C63–C73	1.433(4)	1.420(4)
C47–C49	1.445(4)	1.439(3)	C53–C63	1.432(4)	1.429(3)
C33–C49	1.475(4)	1.451(3)	C53–C55	1.447(4)	1.433(3)
C33–C38	1.442(4)	1.436(3)	C63–C86	1.441(4)	1.432(3)

C33–C40	1.435(4)	1.424(3)	C86–C91	1.388(4)	1.386(4)
C40–C55	1.418(4)	1.421(3)	C82–C91	1.416(4)	1.403(4)
C43–C55	1.442(4)	1.423(3)	C74–C82	1.437(4)	1.429(3)
C43–C75	1.442(4)	1.423(3)	C53–C74	1.421(4)	1.413(4)
C61–C75	1.453(4)	1.427(3)	C59–C74	1.445(4)	1.431(3)
C61–C64	1.419(4)	1.426(3)	C59–C71	1.420(4)	1.418(3)
C51–C64	1.443(4)	1.432(3)	C40–C71	1.447(4)	1.426(3)
C51–C65	1.421(4)	1.415(3)	C45–C71	1.415(4)	1.403(3)
C43–C65	1.443(4)	1.433(3)	C45–C77	1.377(4)	1.374(3)
C61–C67	1.412(4)	1.403(3)	C77–C87	1.408(4)	1.405(4)
C67–C69	1.380(4)	1.369(3)	C83–C87	1.387(4)	1.378(4)
C69–C81	1.419(4)	1.403(3)	C59–C83	1.424(4)	1.414(3)
C81–C85	1.385(4)	1.384(3)	C43–C75	1.422(4)	1.423(3)
C64–C85	1.417(4)	1.409(3)	C49–C75	1.435(4)	1.423(3)

* The C–C bonds are shown in red/blue when the distances are shorter/longer in $\mathbf{1}^{5-}$ compared to the distance in $\mathbf{1}^{4-}$.

VI. Computational Details

Geometry optimizations were performed using the ORCA 5.0.4 suite.[10] Structures without rubidium atoms were optimized at the PBE0/def2-TZVP level of theory.[11, 12] The Zeroth Order Regular Approximation (ZORA)[13] (with the corresponding basis set ZORA-def2-SVP for carbon/hydrogen and SARC-def2-TZVP for rubidium[14]) was used if the structure contained rubidium atoms. Structures containing the crown ethers were optimized using the PBE[15] (instead of PBE0) density functional. Final energies were evaluated using the PBE0 functional and def2-TZVP basis set. In all cases, we adopted the D3-BJ dispersion correction scheme as proposed by Grimme.[16] For efficient evaluation of Coulomb- and exchange integrals, the RIJ-COSX approximation was used.[17, 18]

Transition states and activation barriers were determined from nudged-elastic-band (NEB) calculations followed by transition state optimizations based on the eigenvector-following algorithm.[19] We always checked that the found transition mode corresponds to the expected motion.

Time-dependent DFT (TD-DFT) calculations were carried out using the TMHF functional[20] as implemented in Turbomole 7.6 together with the def2-TZVP basis set for all atoms.

In all calculations, the solvent was modeled using the Conductor-like Polarizable Continuum Model[21] (CPCM, as implemented in ORCA) or Conductor-like Screening Model[22] (COSMO, as implemented in Turbomole, with the screening function $f = \frac{\epsilon - 1}{\epsilon}$) with parameters for THF (ε_r = 7.25, n_{frac} = 1.4070, R_{solv} = 1.3 Å).

VII. Computational Results

Table S5. Energy differences between the boat and chair conformations of $\mathbf{1}^{n-}$, $Rb^+_4(\mathbf{1}^{n-})$ and $\{Rb^+(18\text{-crown-6})\}_4(\mathbf{1}^{n-})$, computed at the PBE0-D3/def2-TZVP (incl. C-PCM(THF)) level of theory, ΔE = E(chair) – E(boat) in kcal/mol. All structures were allowed to relax completely.

	**1**	$\mathbf{1}^{-}$	$\mathbf{1}^{2-}$	$\mathbf{1}^{3-}$	$\mathbf{1}^{4-}$	$\mathbf{1}^{5-}$	$\mathbf{1}^{6-}$
without Rb^+	1.9	2.1	2.2	2.4	2.1	1.9	1.8
with 4 Rb^+	N/A	N/A	N/A	N/A	-1.1	-0.8	-0.4
with 4 $\{Rb^+(18\text{-crown-6})\}$	N/A	N/A	N/A	N/A	-3.0	-2.6	-2.8

Table S6. Relative energies with respect to the boat conformation of the transition states and intermediate for the interconversion of **1**$^{n–}$ (= **HPH**$^{n–}$) from boat to chair conformation, computed at the PBE0-D3/def2-TZVP (incl. C-PCM(THF)) level of theory. See Figure S20 for structures and numbering of the transition states.

	**1**	**1**$^{–}$	**1**$^{2–}$	**1**$^{3–}$	**1**$^{4–}$	**1**$^{5–}$	**1**$^{6–}$
TS 1	12.7	12.2	11.6	11.3	10.9	10.9	10.6
intermediate	5.5	5.3	4.8	4.6	5.2	5.2	5.8
TS 2	12.0	11.6	11.1	10.8	10.2	10.1	9.7

Table S7. Frontier orbital energies and HOMO-LUMO gaps (in eV; level: PBE0-D3/def2-TZVP, C-PCM(THF)) of the boat and chair conformations of **1**$^{n–}$.

	**1**	**1**$^{–}$	**1**$^{2–}$	**1**$^{3–}$	**1**$^{4–}$	**1**$^{5–}$	**1**$^{6–}$
boat							
ε(HOMO)	-5.455	-3.284	-2.554	-1.422	-0.771	-0.432	-0.129
ε(LUMO)	-2.724	-1.975	-0.957	-0.647	-0.286	0.372	1.138
gap	2.731	1.309	1.598	0.775	0.485	0.804	1.267
chair							
ε(HOMO)	-5.465	-3.272	-2.549	-1.414	-0.774	-0.444	-0.136
ε(LUMO)	-2.720	-1.977	-0.957	-0.665	-0.294	0.372	1.126
gap	2.745	1.295	1.592	0.749	0.480	0.816	1.262

Table S8. Energy differences between the boat and chair conformations of $\mathbf{1}^{n-}$ and $Rb^+_4(\mathbf{1}^{n-})$, computed at the PBE0-D3/def2-TZVP (incl. C-PCM(THF)) level of theory, ΔE = E(chair) – E(boat) in kcal/mol. The structures were obtained from the relaxed $\{Rb^+(18\text{-crown-}6)\}_4(\mathbf{1}^{n-})$ geometry by removing the $\{Rb^+(18\text{-crown-}6)\}$ or (18-crown-6) moieties, respectively, *without* subsequent geometry optimization.

	$\mathbf{1}^{4-}$	$\mathbf{1}^{5-}$	$\mathbf{1}^{6-}$
without $\{Rb^+(18\text{-crown-}6)\}$	3.0	3.2	4.3
with 4 Rb^+,<br>without (18-crown-6)	-7.9	-8.5	-8.5

Table S9. Molecular lengths **L** and depths **D** (as defined in the paper, in Å) as obtained from geometry optimizations of the boat conformations of $\mathbf{1}^{n-}$, computed at the PBE0-D3/def2-TZVP (incl. C-PCM(THF)) level of theory.

	**1**	$\mathbf{1}^{-}$	$\mathbf{1}^{2-}$	$\mathbf{1}^{3-}$	$\mathbf{1}^{4-}$	$\mathbf{1}^{5-}$	$\mathbf{1}^{6-}$
**L**	16.378	16.451	16.551	16.582	16.534	16.614	16.612
**D**	3.042	2.974	2.834	2.892	3.004	2.959	3.012

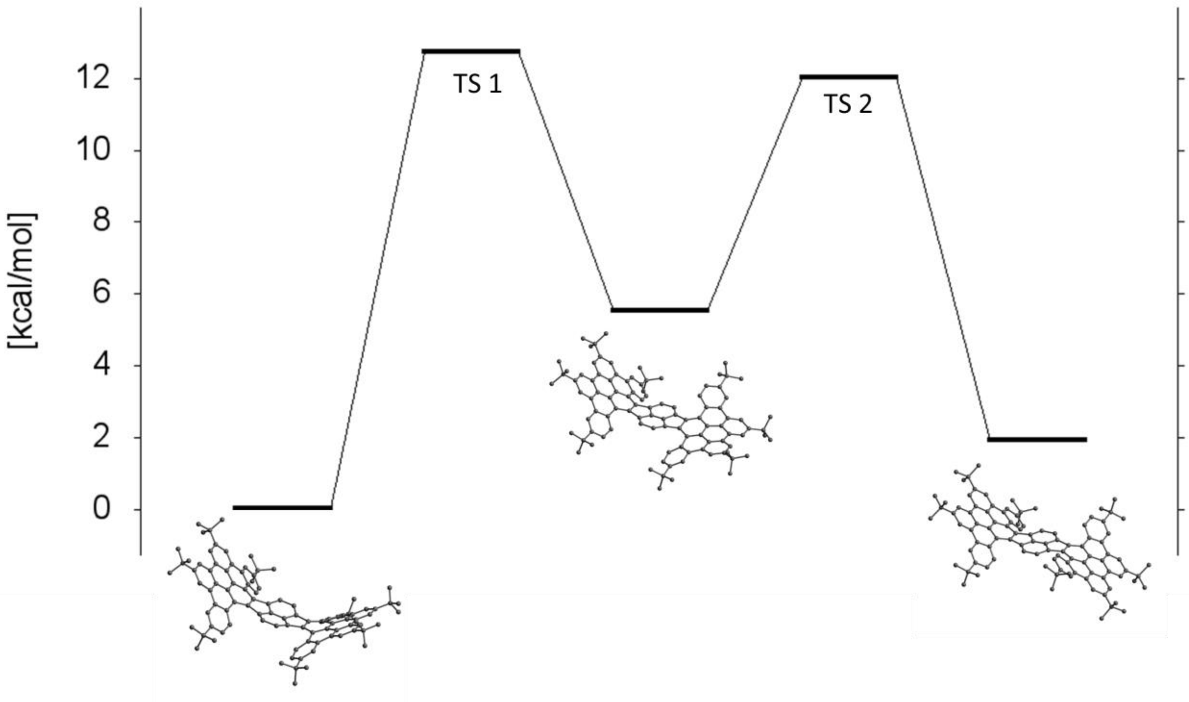

Figure S22. Schematic reaction path from the boat into the chair conformation, via an intermediate conformation, shown for neutral **HPH** (hydrogens omitted). The negatively charged species also follow this reaction path qualitatively; see Tables S5, S6 for the energies.

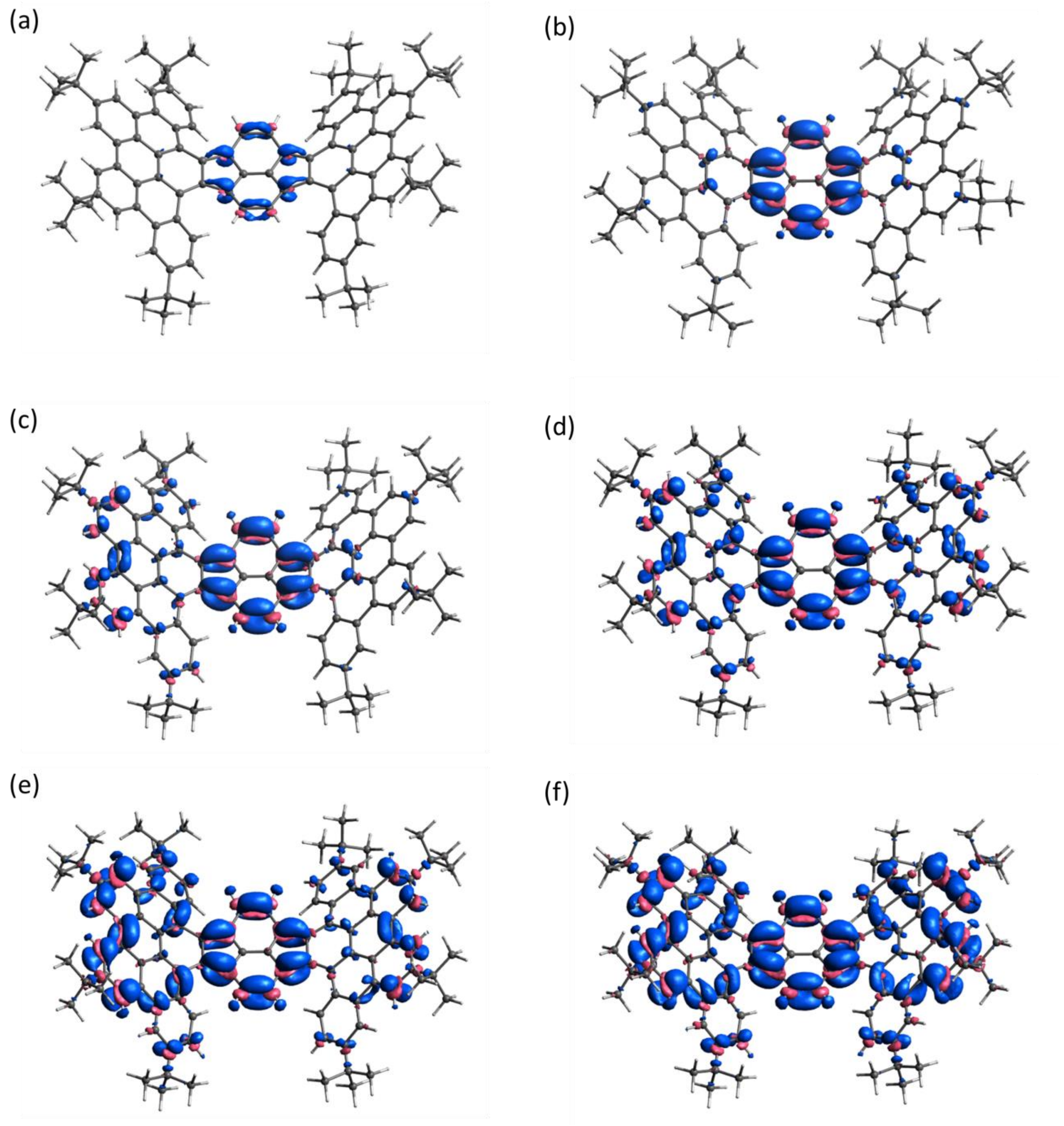

Figure S23. Calculated electron density differences of the negatively charged **1**$^{n-}$ (= **HPH**$^{n-}$) with respect to neutral **HPH**, boat conformation. (a) **HPH**$^{-}$, (b) **HPH**$^{2-}$, (c) **HPH**$^{3-}$, (d) **HPH**$^{4-}$, (e) **HPH**$^{5-}$, (f) **HPH**$^{6-}$. Throughout, iso-contours of ± 0.003 e/bohr^3 are plotted. Electron density differences of the chair conformation are very similar.

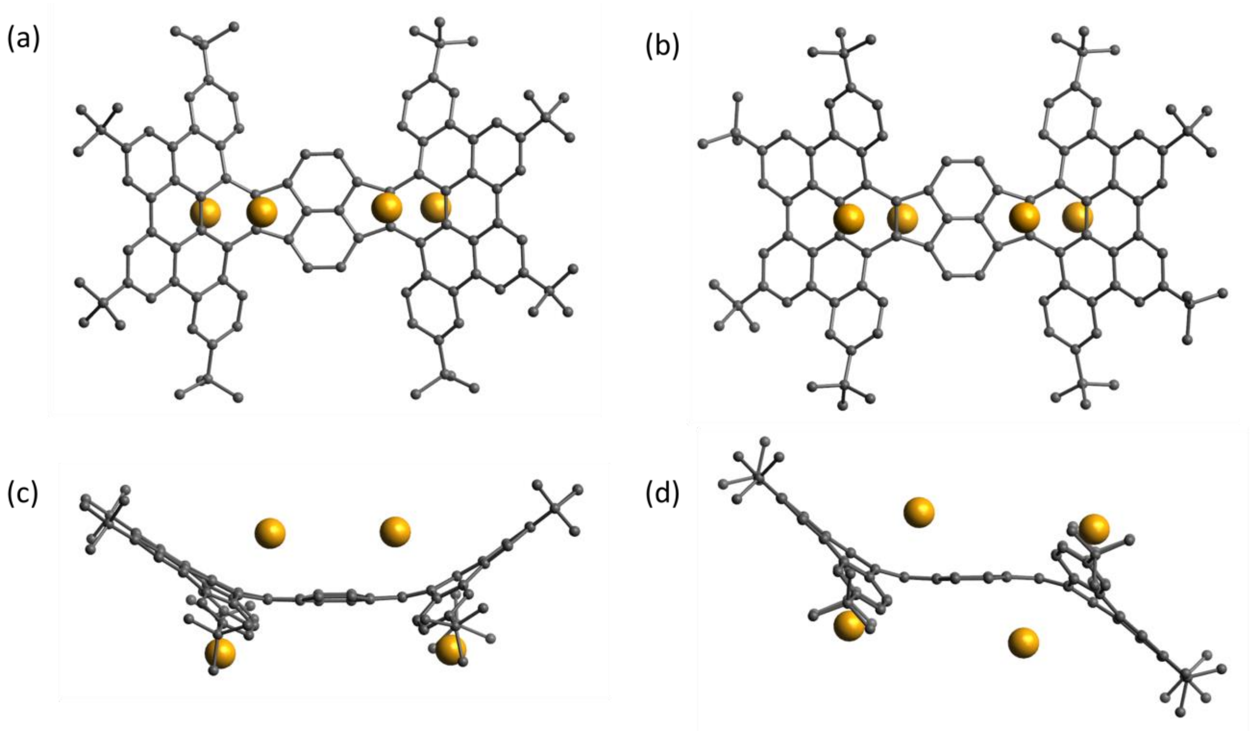

Figure S24. Fully relaxed Rb^+ arrangements in $Rb^+_4(\mathbf{1}^{n-})$ (n = 4, 5, 6) without 18-crown-6 as obtained from geometry optimizations. (Shown are the structures of $\mathbf{1}^{4-}$, but $\mathbf{1}^{5-}$ and $\mathbf{1}^{6-}$ look very similar.) (a) Top view boat conformation, (b) top view chair conformation, (c) side view boat conformation, (d) side view chair conformation. Hydrogen atoms are omitted for clarity.

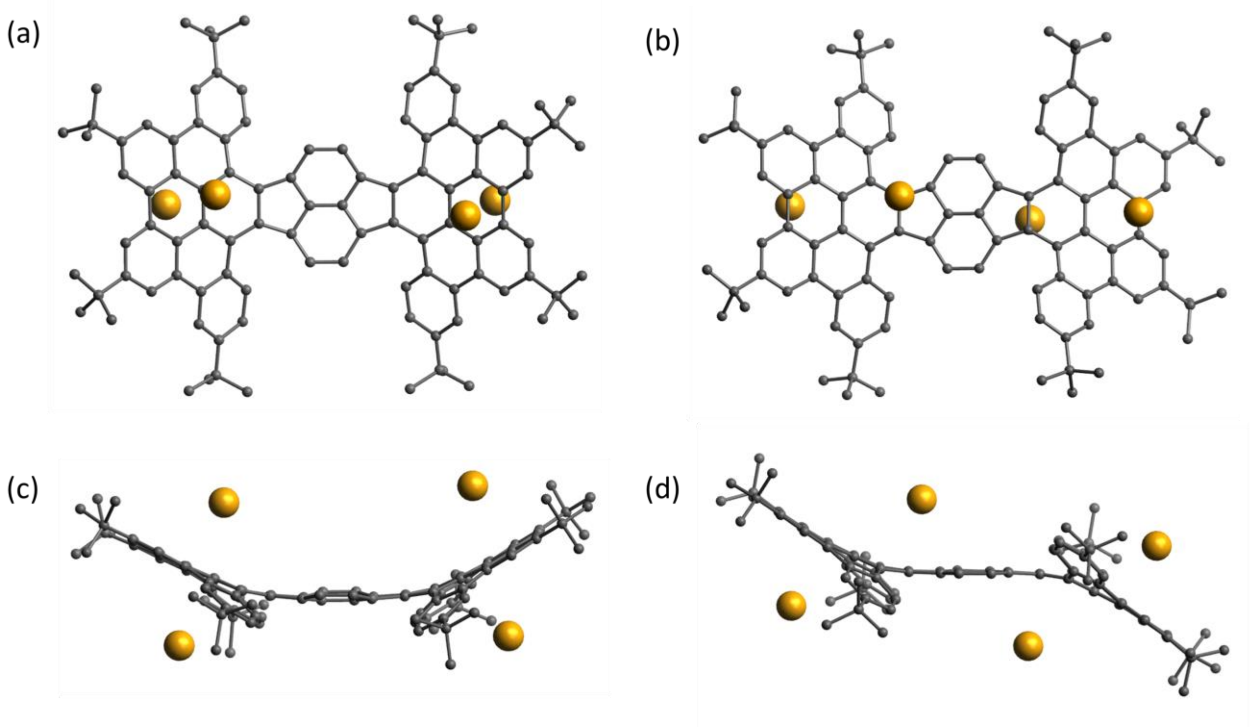

Figure S25. Rb^+ arrangements in $\{Rb^+(18\text{-crown-}6)\}_4(\mathbf{1}^{n-})$ (n = 4, 5, 6) as obtained from geometry optimizations. (Shown are the structures of $\mathbf{1}^{4-}$, but $\mathbf{1}^{5-}$ and $\mathbf{1}^{6-}$ look very similar.) (a) Top view boat conformation, (b) top view chair conformation, (c) side view boat conformation, (d) side view chair conformation. The crown ethers and hydrogen atoms are omitted for clarity.

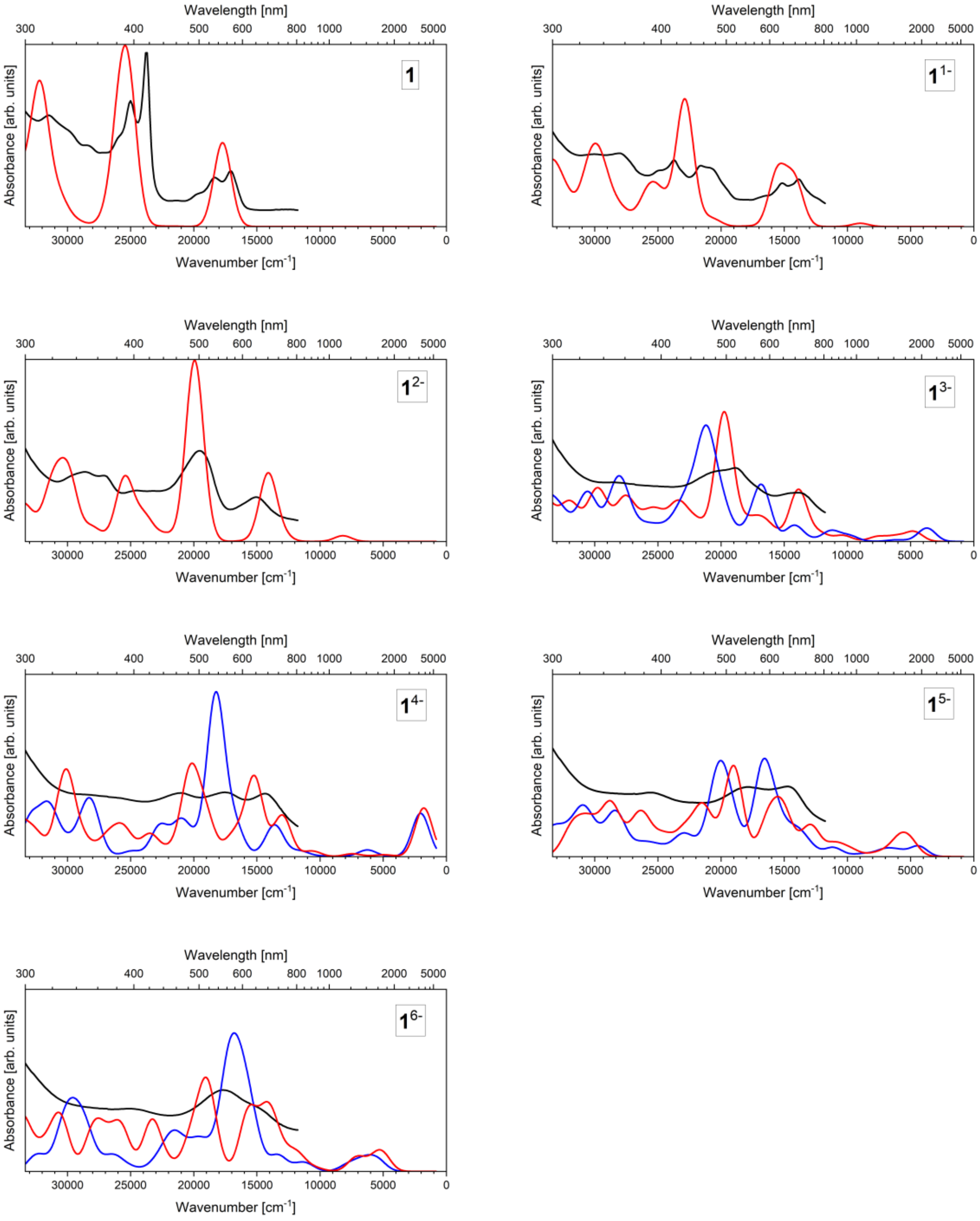

Figure S26. Comparison of experimental (Rb/**1** in THF, *cf.* Fig. **S4**; black) and simulated UV-vis spectra of $\mathbf{1}^{n-}$ (n = 0 to 6, boat conformation; red) and $Rb^{+}_{2}(\mathbf{1}^{3-})$ or $Rb^{+}_{4}(\mathbf{1}^{n-})$ (n = 4, 5, 6, boat conformation, without crown ethers; blue) on the TD-DFT (TMHF) level (incl. an implicit solvent model for THF). Spectra are calculated for the most stable geometry and are shifted by -0.2 eV, respectively.

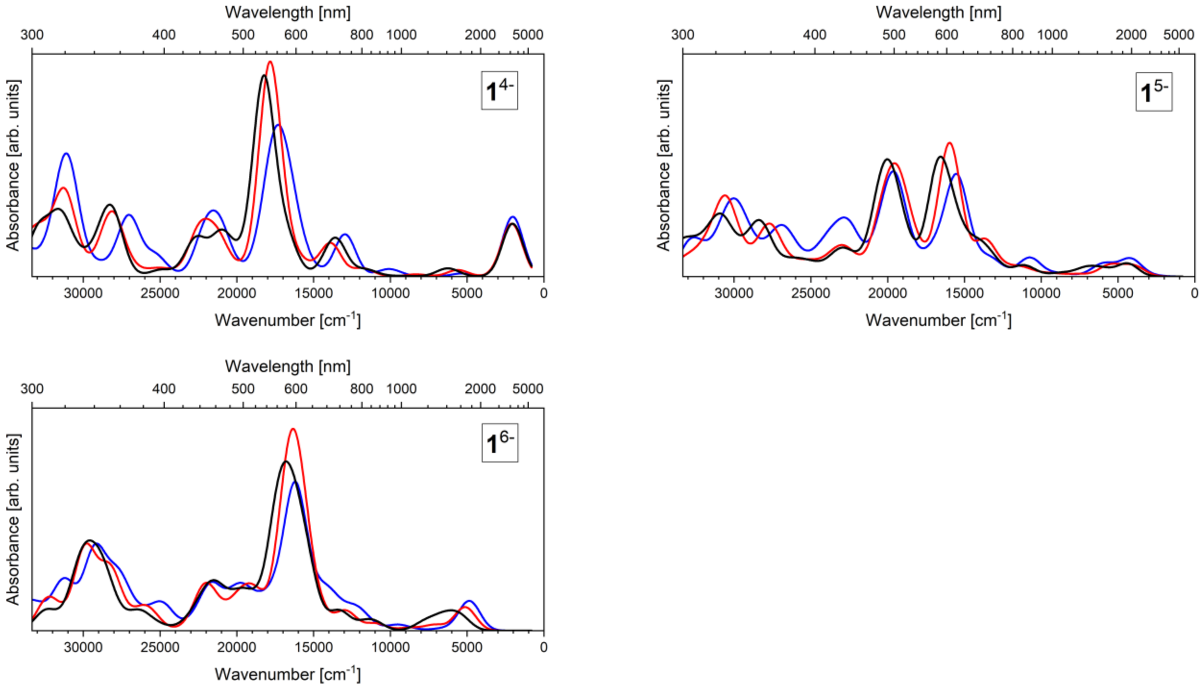

Figure S27. Comparison of simulated UV-vis spectra of $Rb^+{}_4(\mathbf{1}^{n-})$ (n = 4, 5, 6, without crown ethers) on the TD-DFT (TMHF) level (incl. an implicit solvent model for THF) for different conformations and Rb^+ arrangements: most stable Rb^+ arrangement (boat conformation; black), most stable Rb^+ arrangement (chair conformation; red), Rb^+ arrangement as in $\{Rb^+(18\text{-crown-}6)\}_4(\mathbf{1}^{n-})$ (chair conformation; blue). Spectra are shifted by -0.2 eV, respectively.

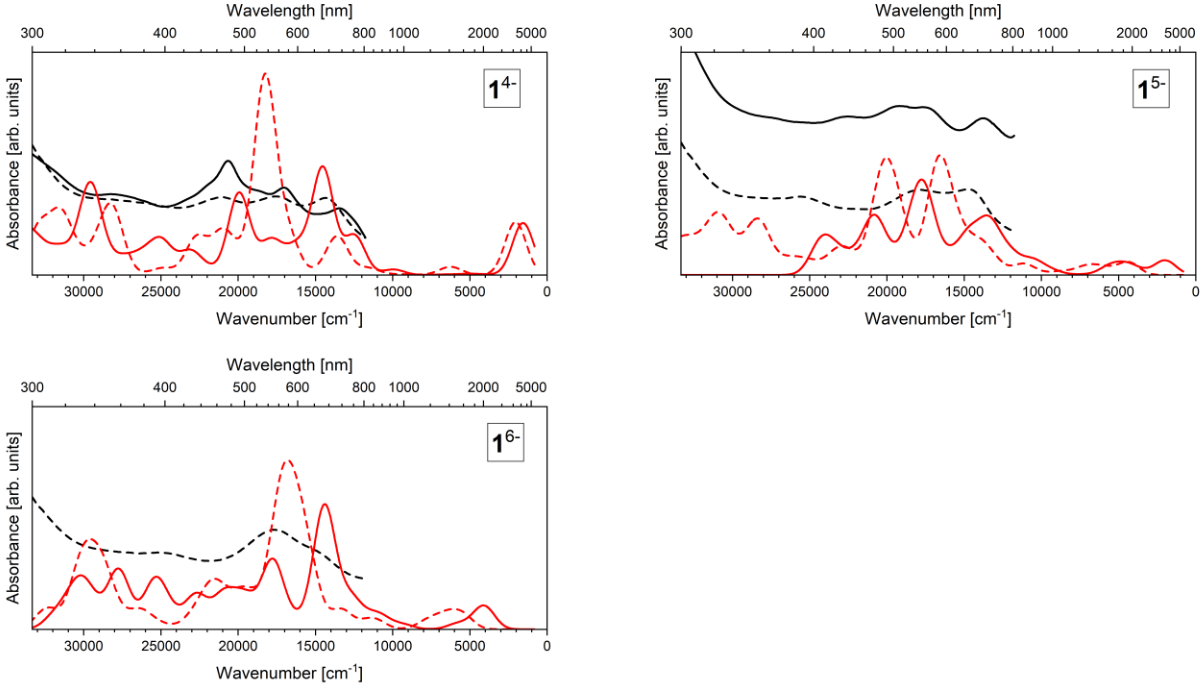

Figure S28. Comparison of experimental UV-vis spectra with (*cf.* Fig. **S3**, black solid) / without (*cf.* Fig. **S4**, black dashed) 18-crown-6 (black solid) and simulated UV/vis spectra of the most stable conformations of {Rb^+(18-crown-6)}$_4$(**1**$^{n-}$) (chair conformation; red solid) / Rb^+_4(**1**$^{n-}$) (boat conformation; red dashed) on the TD-DFT (TMHF) level (incl. an implicit solvent model for THF). Calculated spectra are shifted by -0.2 eV, respectively.

VIII. References

(1) Kozhemyakina, N. V.; Nuss, J.; Jansen, M. Demonstration of the “Break-and-Seal” Approach to Fullerides of Complex Cations at the Example of KC60(THF)5·2THF. *Z. Für Anorg. Allg. Chem.* **2009**, *635* (9–10), 1355–1361.

(2) Bergner, J.; Walla, C.; Rominger, F.; Dreuw, A.; Kivala, M. Inducing Curvature to Pyracylene upon π-Expansion. *Chem. – Eur. J.* **2022**, *28* (47), e202201554.

(3) SAINT; Part of Bruker APEX3 Software Package (Version 2017.3-0): Bruker AXS. **2017**.

(4) SADABS; Part of Bruker APEX3 Software Package (Version 2017.3-0): Bruker AXS. **2017**.

(5) Rigaku Corporation. Rigaku Oxford Diffraction, CrysAlisPro Software System, Version 1.171.43.92a. **2023**.

(6) Rigaku Oxford Diffraction. SCALE3 ABSPACK; A Rigaku Oxford Diffraction Program (1.0.11,Gui:1.0.7) (C). **2005**.

(7) Sheldrick, G. M. SHELXT – Integrated Space-Group and Crystal-Structure Determination. *Acta Crystallogr. Sect. Found. Adv.* **2015**, *71* (1), 3–8.

(8) Sheldrick, G. M. Crystal Structure Refinement with SHELXL. *Acta Crystallogr. Sect. C Struct. Chem.* **2015**, *71* (1), 3–8.

(9) Dolomanov, O. V.; Bourhis, L. J.; Gildea, R. J.; Howard, J. A. K.; Puschmann, H. OLEX2: A Complete Structure Solution, Refinement and Analysis Program. *J. Appl. Crystallogr.* **2009**, *42* (2), 339–341.

(10) a) Neese, F. The ORCA program system. *WIREs Comput. Mol. Sci.* **2012**, *2*, 73–78; b) Neese, F. Software update: The ORCA program system – Version 5.0. *WIREs Comput. Mol. Sci.* **2022**, *12*, e1606.

(11) Adamo, C.; Barone, V. Toward reliable density functional methods without adjustable parameters: The PBE0 model. *J. Chem. Phys.* **1999**, *110*, 6158–6170.

(12) Weigend, F.; Ahlrichs, R. Balanced basis sets of split valence, triple zeta valence and quadruple zeta valence quality for H to Rn: Design and assessment of accuracy. *Phys. Chem. Chem. Phys.* **2005**, *7*, 3297–3305.

(13) van Lenthe, E.; Baerends, E. J.; Snjijyders, J. G. Relativistic regular two- component Hamiltonians. *J. Chem. Phys.* **1993**, *99*, 4597–4610.

(14) Rolfes, J. D.; Neese F.; Pantazis D. A. All-electron scalar relativistic basis sets for the elements Rb-Xe. *J. Comput. Chem.* **2020**, *41*, 1842–1849.

(15) Perdew J. P.; Burke K.; Ernzerhof M. Generalized Gradient Approximation Made Simple. *Phys. Rev. Lett.* **1996**, *77*, 3865–3868; Erratum *Phys. Rev. Lett.* **1997**, *78*, 1396.

(16) a) Grimme, S.; Antony, J.; Ehrlich S.; Krieg, H. A consistent and accurate ab initio parametrization of density functional dispersion correction (DFT-D) for the 94 elements H-Pu. *J. Chem. Phys.* **2020**, *132*, 154104; b) Grimme, S.; Ehrlich, S.; Goerigk, L. Effect of the damping function in dispersion corrected density functional theory. *J. Comput. Chem.* **2011**, *32*, 1456–1465.

(17) a) Neese, F,; Wennmohs, F.; Hansen, A.; Becker, U. Efficient, approximate and parallel Hartree–Fock and hybrid DFT calculations. A ‘chain-of-spheres’ algorithm for the Hartree–Fock exchange. *Chem. Phys.* **2009**, *356*, 98–109; b) Helmich-Paris, B.; de Souza, B.; Neese, F.; Izsák, R. An improved chain of spheres for exchange algorithm. *J. Chem. Phys.* **2021**, *155*, 104109.

(18) a) Eichkorn, K.; Treutler, O.; Öhm, H.; Häser, M.; Ahlrichs R. Auxiliary basis sets to approximate Coulomb potentials. *Chem. Phys. Lett.* **1995**, *242*, 652–660; b) Weigend, F. Accurate Coulomb-fitting basis sets for H to Rn. *Phys. Chem. Chem. Phys.* **2006**, *8,* 1057–1065.
(19) Ásgeirsson, V.; Birgisson, B. O.; Bjornsson, R.; Becker, U.; Neese, F.; Riplinger, C.; Jónsson, H. Nudged Elastic Band Method for Molecular Reactions Using Energy-Weighted Springs Combined with Eigenvector Following *J. Chem. Theo. Comput.* **2021**, *17*, 4929–4945
(20) Holzer, C.; Franzke, Y. J. A local hybrid exchange functional approximation from first principles. *J. Chem. Phys.* **2022**, *157,* 034108.
(21) a) Barone, V.; Cossi, M. Quantum Calculation of Molecular Energies and Energy Gradients in Solution by a Conductor Solvent Model. *J. Phys. Chem. A* **1998**, *102,* 1995–2001; b) Garcia-Rates, M.; Neese, F. Effect of the solute cavity on the solvation energy and its derivatives within the framework of the Gaussian charge scheme. *J. Comput. Chem.* **2020**, *41*, 922–939.
(22) Klamt, A.; Schüürmann, G. COSMO: a new approach to dielectric screening in solvents with explicit expressions for the screening energy and its gradient. *J. Chem. Soc. Perkin Trans. 2* **1993**, *2,* 799–805.
